# Food categories contributions to energy and nutrient intakes among youth adhering to vegan, lacto-ovo-vegetarian, pescatarian or omnivorous diets

**DOI:** 10.1007/s00394-026-04128-y

**Published:** 2026-10-31

**Authors:** Isabelle Mulkerrins, Anine Christine Medin, Synne Groufh-Jacobsen, Claire Margerison, Christel Larsson

**Affiliations:** 1 Department of Food and Nutrition, and Sport Science, Faculty of Education, University of Gothenburg, P.O. Box 300, Gothenburg, SE-405 30 Sweden; 2 Department of Nutrition and Public Health, Faculty of Health and Sport Sciences, University of Agder, Universitetsveien 25, Kristiansand, 4630 Norway; 3 Department of Health Sciences, Oslo New University College, Oslo, Norway; 4 Institute for Physical Activity and Nutrition, Deakin University, Geelong, Australia

**Keywords:** Plant-based, Dietary intake, Food sources, Nutrients, Youth

## Abstract

**Purpose:**

When adopting plant-based diets, animal-sourced foods need to be replaced with nutritionally adequate plant-based alternatives. However, limited studies have assessed which foods provide key nutrients in plant-based diets among youth. This study investigated the contribution of food categories to intakes of energy and nutrients among youth adhering to vegan, lacto-ovo-vegetarian, pescatarian or omnivorous diets in Sweden.

**Methods:**

Dietary intakes of 16–24 year-olds (60 vegans, 59 lacto-ovo-vegetarians, 55 pescatarians, 61 omnivores) were assessed using four 24-h dietary recalls. The contribution of each food category (*n* = 24) to average intakes of energy, macro-, and micronutrients were calculated and investigated.

**Results:**

With increasing exclusion of animal-sourced food categories from the diet, a greater proportion of youths’ intakes of protein, iron, calcium, selenium, and zinc came from meat analogs, legumes, and cereals and grain products. Similarly, a greater proportion of intakes of vitamins (D, B_2_, B_12_) and calcium came from dairy alternatives (highest among vegans). Nevertheless, average intakes of vitamin D, B_12_, calcium (vegans only), and selenium were lower among vegans and lacto-ovo-vegetarians than among omnivores. In contrast, replacing animal-sourced foods with plant-based alternatives led to higher intakes of fiber and lower intakes of saturated fat.

**Conclusion:**

The more animal-sourced food categories that were excluded from the diet, the more youths’ relied on cereals and grain products, meat analogs, dairy alternatives (fortified), and legumes as sources of energy and nutrients. Particular attention to vitamin D, B_12_, selenium, and calcium (vegans only) and their food sources may be needed when planning vegan and lacto-ovo-vegetarian diets.

**Supplementary Information:**

The online version contains supplementary material available at https://doi.org/10.1007/s00394-026-04128-y.

## Introduction

There is a need for a global shift towards diets which are characterised by higher intakes of fruits, vegetables, berries, whole grains, legumes, nuts and seeds, and lower intakes of animal-sourced foods, particularly red and processed meat, to support long-term human health and environmental sustainability [1, 2]. Heightened awareness of the environmental and health benefits of plant-based diets (e.g., vegan, lacto-ovo-vegetarian, pescatarian) has contributed to an increased adherence to these dietary practices in Western countries in recent years [3, 4].

Youth represent a population group leading the transition towards reducing meat consumption and adopting plant-based dietary patterns [3, 4]. For example, in Sweden, the prevalence of lacto-ovo-vegetarian and vegan diets was 8% among individuals aged 16–34 years, compared with only 3% among those aged 56–79 years, in year 2024 [4]. Despite this, youth remain relatively understudied in nutrition research. The youth stage of life [5] is characterised by increasing autonomy over food choices, and dietary habits established during this pivotal period may persist into adulthood. At the same time, nutrition and health are not always the primary factors influencing youths´ food choices [6]. This may have implications for long-term health, as youth continue to have relatively high requirements for several nutrients, including calcium and iron among menstruating females [2].

Previous studies among adults as well as children and adolescents have reported lower average intakes (from food sources only) of protein, vitamins (B_2_, B_12_, D), and iodine among vegans and lacto-ovo-vegetarians compared with omnivores, as well as lower average intakes of calcium among vegans compared to omnivores [5–7]. At the same time, nutritional challenges are not limited to plant-based dietary patterns. Among Swedish adolescents, inadequate vitamin D intakes have previously been reported, while their average saturated fatty acid (SFA) intakes exceed dietary recommendations and dietary fibre intakes fall below recommendations [8]. Nevertheless, vegan and lacto-ovo-vegetarian diets have also been associated with higher intakes of dietary fibre, folate, vitamin C and iron, and lower intakes of SFA than omnivorous diets [5–7]. Together, these findings indicate that plant-based diets may influence nutrient intakes in both favourable and unfavourable ways.

Well-planned plant-based diets, including lacto-ovo-vegetarian and vegan diets, can be nutritionally adequate and are supported by several food and health organisations, including the Swedish Food Agency [9–12]. However, the nutritional adequacy of a diet depends on the foods consumed, the quantity of foods consumed, and an individual’s specific nutrient requirements. For some nutrients, including vitamins D and B_12_, there are few plant-based food sources which naturally contain these nutrients, and therefore fortified foods and/or supplements ought to be consumed [9, 11]. Dietary guidelines for vegan and lacto-ovo-vegetarian diets emphasise that meat and fish should be replaced with nutritionally appropriate food sources including plant-based foods, fortified foods, and supplementation may also be necessary, particularly for individuals following vegan diets [9, 11, 13].

The Swedish food landscape has changed considerably over the past decade, including an increase in the availability and variety of plant-based alternatives to meat, fish, and dairy products, as well as plant-based ready meals and discretionary foods. These products vary in their nutritional content depending on main ingredients and fortification practices [14–16]. Globally, fortification practices differ [17, 18]. In Sweden, plant-based meat analogs are not commonly fortified with vitamins and minerals which are typically found in meat and fish, e.g., vitamin B_12_ and vitamin D [14]. However, in Sweden, plant-based milk and yoghurt alternatives are mandatorily fortified with vitamin D and are often voluntarily fortified with B_2_, B_12_ and calcium [16, 19], while margarines are mandatorily fortified with vitamins A and D [19]. Therefore, in Sweden several fortified plant-based food groups may now make important contributions to key nutrients, such as vitamin D and B_12_, that have traditionally been obtained from animal-sourced foods.

Despite changes in food availability, limited evidence exists regarding the food sources contributing to energy and nutrient intakes among individuals consuming plant-based diets [20–22]. In Sweden, this was last examined in a small study of 16–20 year-olds in the late 1990s (*n* = 30 vegans; *n* = 30 omnivores) [21]. Understanding the contribution of different food categories to energy and nutrient intakes can provide insight into the role of different food sources, including fortified foods, in supporting the nutritional adequacy of modern-day plant-based diets and may help inform dietary guidance and recommendations specific to plant-based diets. This study investigated the contribution of food categories to average intakes of energy and nutrients among youth adhering to vegan, lacto-ovo-vegetarian, pescatarian or omnivorous diets in Sweden.

## Materials and methods

### Study design and eligibility criteria

This cross-sectional study is part of a larger mixed-methods research project named *VeggiSkills-Sweden* [23, 24]. The overall aim of the project was to assess the nutritional consequences of consuming plant-based diets of differing strictness (vegan, lacto-ovo-vegetarian, pescatarian) or omnivorous diets among youth in Sweden. We have previously assessed and reported on the usual intakes of food groups, energy, and nutrients, and the nutritional status among the study sample [23, 24]. However, little is known about how food sources contribute to dietary intakes among current youths’ consuming plant-based diets or omnivorous diets.

Healthy youth (*n* = 244) aged 16–24 years were recruited by convenience and snowball sampling and asked to partake in the research project. To ensure that all participants could provide their own informed consent, in accordance with ethical regulations in Sweden, the age range was set as 16–24 years (as individuals aged 16 years and older can provide their own informed consent). Participants were recruited between December 2022 and January 2024 from Gothenburg, Sweden and other nearby municipalities. Briefly, all participants visited a research facility at the University of Gothenburg once to complete several measurements on site. This included completing a 24-hour dietary recall (24HDR), providing blood samples and a urine sample, measuring their anthropometrics and body composition, and completing a web-based questionnaire. Following the visit, participants were asked to complete three more 24HDRs (non-consecutive) on their own during the following weeks. Minimum of one, and maximum of two, 24HDR were to be from a weekend day.

To be eligible to participate they had to be, ‘aged between 16–24 years’; ‘be healthy, with no chronic or acute illness’; ‘not be pregnant, lactating, or have children’; ‘have adhered to their current dietary practice (vegan, lacto-ovo-vegetarian, pescatarian, omnivore) for a minimum of six months prior with no plan to alter their current dietary practice’; ‘understand Swedish’; ‘live in Gothenburg, or nearby municipalities’; and ‘agree to physically visit the research facility once to complete several measurements on site’.

### Sample size

The number of participants aimed for in this study was calculated a priori, based on being able to detect differences between two dietary groups, i.e., vegans and omnivores, who have the most contrasting dietary practices, and also with the intent to stratify findings by sex. Calculations were made based on primary outcome (energy intake) and secondary outcomes (intakes of iron, vitamin D and B_12_) of the research project. More details on the sample size calculations have been reported previously [23, 24]. To detect differences in our primary outcome between two groups, with 80% power and a significance level of 5%, and to account for dropouts (30% drop-out rate), we estimated that we would require 60 participants per dietary group. As we planned to recruit youth adhering to four different dietary practices, we aimed to recruit 240 participants to the study.

### Categorization into dietary groups and exclusion of participants

In a web-based questionnaire, participants were asked to select the dietary practice they had followed in the past six months. To categorize participants into dietary groups, their self-reported dietary practice was cross-checked with their responses to a question asking about how often (‘never’, ‘rarely’, ‘sometimes’, or ‘daily’) they had included different animal-sourced foods in their diet in the past six months (i.e., ‘milk and/or dairy products, including in foods and/or cooking’, ‘egg, including in foods and/or cooking’, ‘fish/seafood, including fish products’, ‘poultry and/or poultry products’, ‘red meat and/or red meat products’). They were categorized as vegan if they self-reported a vegan diet and selected ‘never’ to all the animal-sourced foods. They were categorized as lacto-ovo-vegetarian if they self-reported a lacto-, ovo-, or lacto-ovo-vegetarian diet and included eggs and/or dairy, but not fish/seafood or meat (any type). They were categorized as pescatarian if they self-reported a pescatarian diet and included fish, but not meat (any type), and regardless of inclusion of dairy and eggs. They were categorized as omnivore if they self-reported an omnivorous diet and included meat (any type) and at least one other animal-sourced food in their diet.

During this stage of data processing seven participants (six flexitarians, one pescatarian) were excluded due to dietary practice ineligibility, according to inclusion criteria. For more details of how dietary practice ineligibility was confirmed, see previous publications [23, 24].

### Assessment of dietary intake

To assess dietary intake, each participant was asked to conduct four non-consecutive 24HDRs in a web-based dietary assessment program, Nutrition Data (Nutrition Data Sweden AB, Sweden). The first 24HDR was completed on site as an interview following the multiple pass method. The remaining 24HDRs were completed by the participant on random, non-consecutive days the following weeks. The dietary assessment method has been described in detail previously [23, 24]. The dietary assessment program automatically calculated intakes of energy and nutrients using the Swedish Food Composition Database (version 20230613, Swedish Food Agency) or Nutrition Datas’ own Food Composition Database. For each individual, their intakes of food categories, energy, and nutrients were averaged based on the number of recalls completed (between 2 and 4). Two participants who only completed the first 24HDR on site were excluded from data analysis. This ensured consistency with our prior publications, where usual intakes were estimated and at least two days of dietary intake data per participant was required.

In this paper, dietary supplements reported in the 24HDR were excluded from the analyses of dietary intakes since the focus is to investigate the contribution of food sources alone to intakes of energy and nutrients. In the supplementary material, the contributions from food categories *and* dietary supplements to intakes of some key selected vitamins (A, D, B_2_, B_12_) and minerals (iron, calcium, selenium, zinc), stratified by supplement user and dietary practice, are available (Supplementary Tables 1–8).

### Categorization of foods into food categories and disaggregation of composite meals

All items (approximately 2370 unique food items) reported in the 24HDR were manually coded and categorized into broad overall categories (*n* = 24, excluding a category for water and a category for dietary supplements) based on nutritional similarity or culinary use. The food category, cereal and grain products, included both refined grain and whole grain products (e.g., bread, bread products, pasta, noodles, rice, grains, pseudograins, oats, and breakfast cereals). The category ‘vegetables, fruits, and berries’ included processed vegetable products (e.g., coleslaw, beetroot salad) and dried fruits, but did not include fruit juices (separate category). The category plant-based dairy alternatives included all types of plant-based alternatives for milk, yogurt, cream, crème fraiche, and cheese. The category plant-based meat analogs included all types of plant-based meat analogs (e.g., based on soy, pea, oat, mycoprotein etc.) as well as food items such as tofu, tempeh, textured vegetable protein, and vegetarian sandwich toppings. The category legumes included legume-based products such as bean burgers, falafel, and hummus (products which are based on the whole legume). The overall food categorizations were cross-checked and referenced with other similar studies to maintain comparability, as far as possible [20, 25, 26]. See a previous publication for a detailed description and list of foods included in each food category in this study [24].

In the 24HDR, participants were instructed to report their dietary intake with as much details as possible. This included an instruction to report foods as ingredients rather than composite meals when possible. For example hamburger bun, meat patty, tomato, cheese, instead of ‘hamburger’. For food/drink items which were consumed but were not available in the web-based program, participants were instructed to select a nutritionally similar food item available in the program or to report the food item consumed in an open text box. All food items specified in the open text box were entered into the 24HDR by the first author. This process has been described in more detail previously [23, 24]. Of the composite meals reported, most were disaggregated into ingredients by the first author. This was done by using standard recipes. Briefly, if the composite meal reported was a food item from the Swedish Food Composition Database, the recipe specified in the Swedish Food Agency database was used, and quantities of ingredients were scaled according to the quantity of the meal reported as consumed. If the participant provided any specific information about the composite meal, for example brand product name (e.g., Anamma frozen vegochilli), the ingredient list was obtained from online sources, primarily using the brand’s own website. When possible, the ingredient list was used to disaggregate the food into ingredients and aiming to match the recipe to the nutritional content of the specific food item (information obtained from online websites). For composite food items (*n* = 20) where it was not possible to disaggregate into ingredients, such as spring rolls, dumplings, frozen Quorn lasagne, they were categorized into the food category ‘composite meals’.

### Proportion of consumers of different categories of foods

To provide an indication of the prevalence and frequency of consumption of different food categories among youth, the proportion consuming each food category was assessed. This was done by categorizing each participant as a consumer or a non-consumer of the overall food category. In this paper a consumer was defined as having reported intake of the food category in at least one of the 24HDR.

### Contribution of food categories to intakes of energy and nutrients

To calculate the contribution (as a percentage) of each food category to average intakes of energy, macronutrients, fatty acids (saturated, mono- and polyunsaturated, omega-3 and 6), and micronutrients, the mean proportion method was used [27]. For each participant their average intake of each nutrient was calculated i.e., total intake from all their 24HDRs, which was then averaged by dividing by the number of completed recalls, between 2 and 4. Also, the average intake of each nutrient (energy, macronutrients, fatty acids, micronutrients) from the different food categories (*n* = 24) was calculated. Briefly, the total intake of the nutrient from the specific food category was calculated, which was then averaged by dividing by the number of completed recalls. Then, the contribution of the food category to the average intake of the nutrient was calculated. For example: [(average intake of vitamin D from plant-based dairy alternatives/average intake of vitamin D in total)*100]. Then, for each dietary group, the mean of individual percentages was calculated. For each nutrient, food categories providing ≤ 1% of the average intake were summed together and grouped into a ‘miscellaneous’ category.

In this paper, results are presented descriptively for intakes of energy, protein, dietary fiber, and SFA, and for eight key selected micronutrients (vitamins A, D, B_2_, B_12_, iron, calcium, selenium, and zinc). These eight micronutrients were selected since they are considered nutrients of concern when adhering to a plant-based diet, especially a vegan diet [5–7, 9, 13, 28]. Iodine is also considered a micronutrient of concern in a plant-based diet. However, in Sweden, table salt is voluntarily fortified with iodine and is commonly used in households [29], making it a main dietary source. Yet, accurate assessment of salt intake is difficult. Furthermore, iodized salt is included in the nutrient content of various foods (cooked pasta, boiled potatoes) in the Swedish Food Composition Database. Together, this makes it challenging to accurately identify iodine sources in the diet and the contribution of food categories (separate from salt) to iodine intakes. For the same reason, we did not assess the contribution of food categories to sodium intake. For other nutrients, i.e., carbohydrates, free sugars, total fat, mono- and polyunsaturated fatty acids, omega-3 and 6 fatty acids, vitamins (B_1_, B_6_, C, E, K, folate) and minerals (niacin, magnesium, potassium, phosphorous) the results are presented descriptively in figures in the Supplementary Material (Supplementary Figs. 1–8).

### Assessment of sociodemographic variables and anthropometrics

To assess the participants’ sociodemographics (e.g., educational level, parental education level) and dietary habits (e.g., dietary practice, diet duration) they were asked to fill in a web-based questionnaire during their visit at the research facility. For educational level, the response alternatives ‘university less than 3 years’ and ‘university 3 years or longer’ were aggregated into one category: ‘university education’.

To assess their anthropometrics (bodyweight and height), participants were measured during their research facility visit using a wall-mounted stadiometer (Hyssna M, Sweden) and digital scale (Beurer GmbH, Germany). Body mass index (BMI) was calculated as kg/m^2^.

### Statistical analyses

To test for differences between dietary groups in categorical variables, crosstabulations with Chi-Square or Fischer’s exact test was used. Data is presented as numbers and percentages (%).

Normality of continuous variables (age and BMI) was assessed using Q-Q plots and histograms. The data were not normally distributed; however, both parametric (ANOVA) and non-parametric (Kruskal Wallis) tests were performed and showed largely similar results. To correct for multiple analyses, Bonferroni post hoc tests were applied. In this study, results for age and BMI are presented as means ± standard deviations (SD), allowing for comparability of our results with other studies. Data was processed using Microsoft Excel, and analysed using SPSS Version 29 (Chicago, IL, USA). For all tests, an adjusted (two-sided) *p*-value of < 0.05 was accepted as statistically significant.

## Results

### Sample characteristics

The characteristics of participants are presented in Table 1.

**Table 1:** Participant characteristics of youth aged 16–24 year-olds adhering to vegan, lacto-ovo-vegetarian, pescatarian and omnivorous diets (*n* = 235)

	Total sample (*n* = 235)		Vegan (*n* = 60)		Lacto-ovo-vegetarian (*n* = 59)		Pescatarian (*n* = 55)		Omnivore (*n* = 61)		*P*-value
	Mean	SD	Mean	SD	Mean	SD	Mean	SD	Mean	SD	
Age, years^*^	22	2	22	2	22	2	21	2	21	2	0.05
BMI, kg/m^2*^	23	3	22	3	22	3	22	3	23	3	0.07

	Total sample (*n* = 235)		Vegan (*n* = 60)		Lacto-ovo-vegetarian (*n* = 59)		Pescatarian (*n* = 55)		Omnivore (*n* = 61)		*P*-value
	N	%	N	%	N	%	N	%	N	%	
*Sex* ^†^											0.12
Female	183	78	45	75	49	83	47	86	42	69	
Male	52	22	15	25	10	17	8	15	19	31	
*Duration of adherence to dietary practice* ^†^											**< 0.001**
≤ 11 months	15	6	1	2	6	10	5	9	3	5	
1–3 years	44	19	16	27	11	19	10	18	7	12	
3–5 years	59	25	21^a^	35	20^a^	34	13	24	5^b^	8	
5 + years^‡^	117	50	22^a^	37	22^a^	37	27^a^	49	46^b^	75	
*Highest educational level* ^†^											0.85
< 12 years^§^	19	8	4	7	3	5	6	11	6	10	
12 years (high school degree)	110	47	27	45	27	46	25	46	31	51	
University education^||^	106	45	29	48	29	49	24	44	24	39	
*Mothers highest educational level* ^†^											0.75
< 12 years	10	4	3	5	2	3	1	2	4	7	
12 years (high school degree)	34	15	10	17	6	10	10	18	8	13	
University education^||^	191	81	47	78	51	86	44	80	49	80	
*Fathers highest educational level* ^†^											0.75
< 12 years	23	10	5	8	7	12	4	7	7	12	
12 years (high school degree)	58	25	15	25	12	20	12	22	19	31	
University education^||^	154	66	40	67	40	68	39	71	35	57	
*Reported dietary supplements in the 24HDR* ^†,, ¶^	113	48	46^a^	77	31^b^	53	21^b, c^	38	15^c^	25	**< 0.001**

^*^To test for difference between dietary groups, one-way ANOVA with Bonferroni post hoc test was used. ^†^To test for difference between dietary groups, crosstabulations with Pearsons Chi-Square or Fisher’s Exact test was used, with Bonferroni post hoc test to correct for multiple analysis. ^‡^Also includes the response alternative, ‘whole life’. ^§^ Includes participants aged 19 years or younger (16–19 years) who have not had the chance to finish high school. ^||^Includes the response alternative ‘less than 3 years of university education’ and ‘3 years or more of university education’ ^¶^Supplement user is defined as having reported a dietary supplement (any type, e.g., multivitamin or nutrient specific supplement) at least once in the 24-h dietary recalls. Unlike letters (a, b, c) within the same row indicate which dietary groups differ. Adjusted *p*-value of < 0.05 was accepted as statistically significant

The study sample consisted of 235 healthy youth aged 16–24 years (60 vegans, 59 lacto-ovo-vegetarians, 55 pescatarians, 61 omnivores). In the total sample, the majority were female (78%). The proportion of males was somewhat higher in the omnivorous dietary group (31%) compared to the vegan (25%), lacto-ovo-vegetarian (17%), and pescatarian (15%) groups, but it was not significantly different. In the total sample, the mean (± SD) age was 22 ± 2 years, and the mean (± SD) BMI was 23 ± 3 kg/m^2^ which is within the healthy BMI range of 18.5–24.9 kg/m^2^. Mean age and BMI did not significantly differ between the four dietary groups.

### Proportion of youth consuming animal-sourced foods, plant-based meat analogs and dairy alternatives, legumes, and sweets and snack foods

The percentage of youth reporting consumption of each food category, in the 24HDRs, is presented in Table 2.

**Table 2:** Percentage of youth adhering to vegan, lacto-ovo-vegetarian, pescatarian, or omnivorous diets reporting consumption of different food categories (*n* = 235)

Food category	Total sample (*n* = 235)		Vegan (*n* = 60)		Lacto-ovo-vegetarian (*n* = 59)		Pescatarian (*n* = 55)		Omnivore (*n* = 61)		
	*N*	%	*N*	%	*N*	%	*N*	%	*N*	%	*P*-value
Vegetables, fruits, and berries^*^	235	100	60	100	59	100	55	100	61	100	n/a
Cereals and grain products^*^	235	100	60	100	59	100	55	100	61	100	n/a
Legumes^*^	183	78	56^a^	93	47^a^	80	49^a^	89	31^b^	51	**< 0.001**
Nuts and seeds^*^	172	73	50	83	45	76	37	67	40	66	0.10
Potatoes (all types)^*^	190	81	50	83	45	76	46	84	49	80	0.73
Fruit and vegetable juice^*^	128	55	37	62	35	59	28	51	28	46	0.27
Plant-based dairy alternatives^*^	180	77	60^a^	100	50^b^	85	38^b, c^	69	32^c^	53	**< 0.001**
Plant-based meat analogs^*^	181	77	60^a^	100	54^a^	92	52^a^	95	15^b^	25	**< 0.001**
Fats and oils^*^	232	99	59	98	58	98	55	100	60	98	1.00
Dairy produts (all types)^*^	171	73	0^a^	0	56^b^	95	55^b^	100	60^b^	98	**< 0.001**
Egg^*^	115	49	0^a^	0	29^b^	49	41^c^	75	45^c^	74	**< 0.001**
Fish and seafood^*^	76	32	0^a^	0	1^a^	2^†^	36^b^	66	39^b^	64	**< 0.001**
Meat (all types) ^*^	61	26	0	0	0	0	0	0	61	100	n/a
Sweets and snack foods^*^	235	100	60	100	59	100	55	100	61	100	n/a
Protein bars/powders^*^	82	35	25	42	20	34	20	36	17	28	0.46
Jams, marmalades, and compotes^*^	99	42	18	30	25	42	24	44	32	53	0.09
Sauces, dressings, and mayonnaise^*^	219	93	58	97	53	90	52	95	56	92	0.47
Sugar-sweetened beverages^*^	130	55	30	50	26	44	36	66	38	62	0.07
Coffee and tea^*^	175	75	43	72	49	83	40	73	43	71	0.36
Herbs, spices, and condiments^*^	224	95	60	100	56	95	50	91	58	95	0.10

n/a, not applicable

A consumer was defined as having reported intake of food(s) from the food categories in at least one of the 24-h dietary recalls. ^*^To test for difference between dietary groups crosstabulations with Pearsons Chi-Square test or Fisher’s Exact test was used, with Bonferroni post hoc test to correct for multiple analysis. ^†^One participant who self-reported a lacto-ovo-vegetarian diet and reported no consumption of animal-source food groups in the past six months in the web-based questionnaire reported consumption of fish on one occasion. Bolded *p*-values indicate a significant difference between dietary groups, and unlike letters (a, b, c) within the same row indicate between which dietary groups there is a significant difference after correction for multiple analysis. Adjusted *p*-value of < 0.05 was accepted as statistically significant. A detailed description of food items included in each food category has been published elsewhere [24]

Almost all lacto-ovo-vegetarians, pescatarians, and omnivores reported consuming dairy products in the 24HDRs (Table 2). Consumption of egg was less common, and fewer lacto-ovo-vegetarians (49%) reported consuming eggs in the 24HDRs compared to pescatarians (75%) and omnivores (74%), *p* = 0.005. The proportion of consumers of plant-based meat analogs, plant-based dairy alternatives, and legumes differed significantly across dietary groups (all *p* < 0.001), with higher proportions observed among vegans, lacto-ovo-vegetarians, and pescatarians than among omnivores (Table 2). Almost all youth reported consuming sweets and snack foods in the 24HDRs, and the proportion of consumers did not differ significantly between groups.

### Food categories contribution to average intakes of energy, protein, dietary fiber, SFA, and key selected micronutrients

**Fig. 1:**
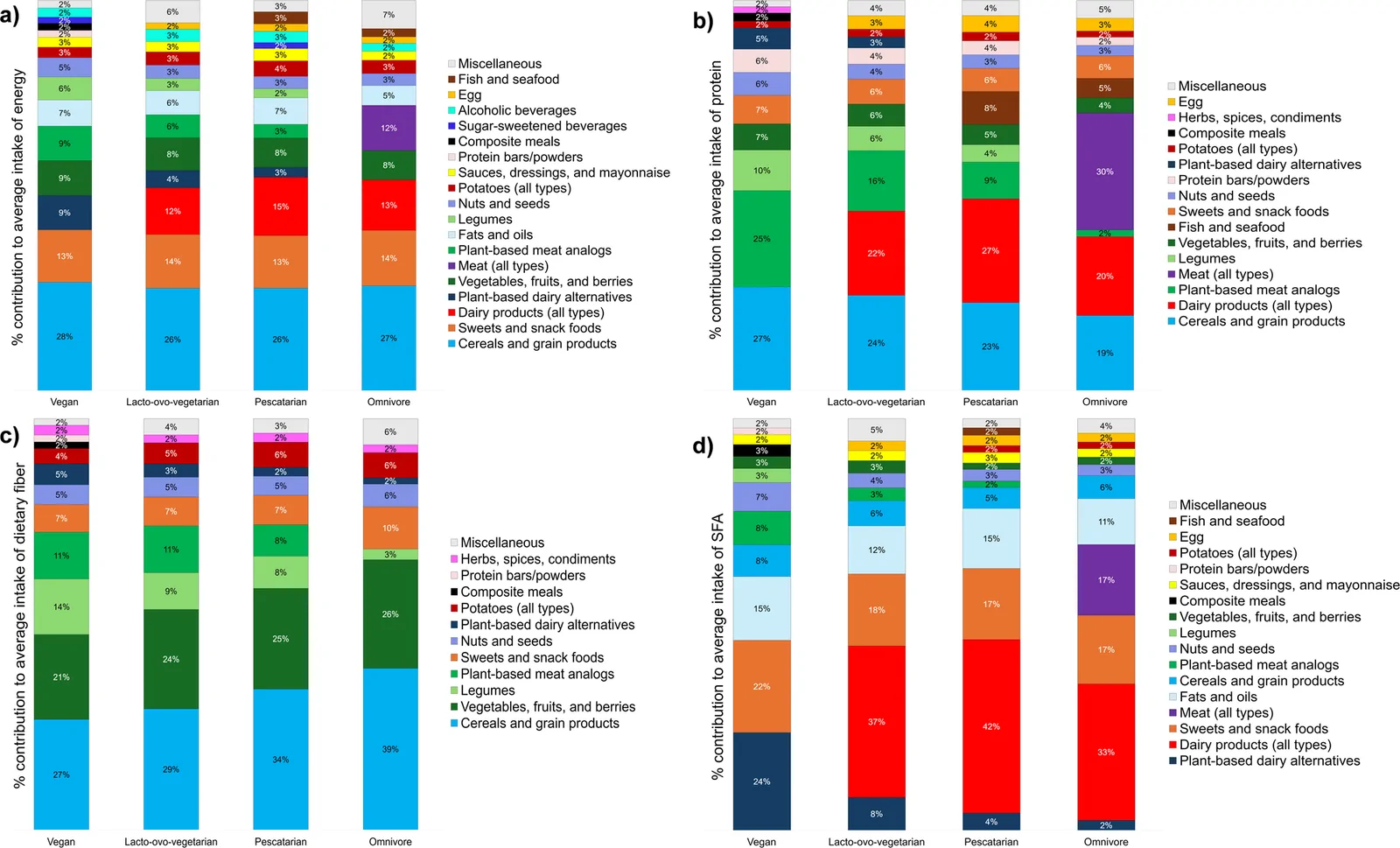
The contribution (%) of different food categories to the average intakes of **a** energy, **b** protein, **c** dietary fiber, and **d** saturated fatty acids (SFA) among youth adhering to vegan, lacto-ovo-vegetarian, pescatarian, or omnivorous diets. The category ‘miscellaneous’ includes all food categories that each contributed ≤ 1% of the average intake, and represents the sum of these categories. The category ‘composite meals’ includes composite foods (e.g., lasagne, spring rolls), where it was not possible to disaggregate into ingredients

**Fig. 2:**
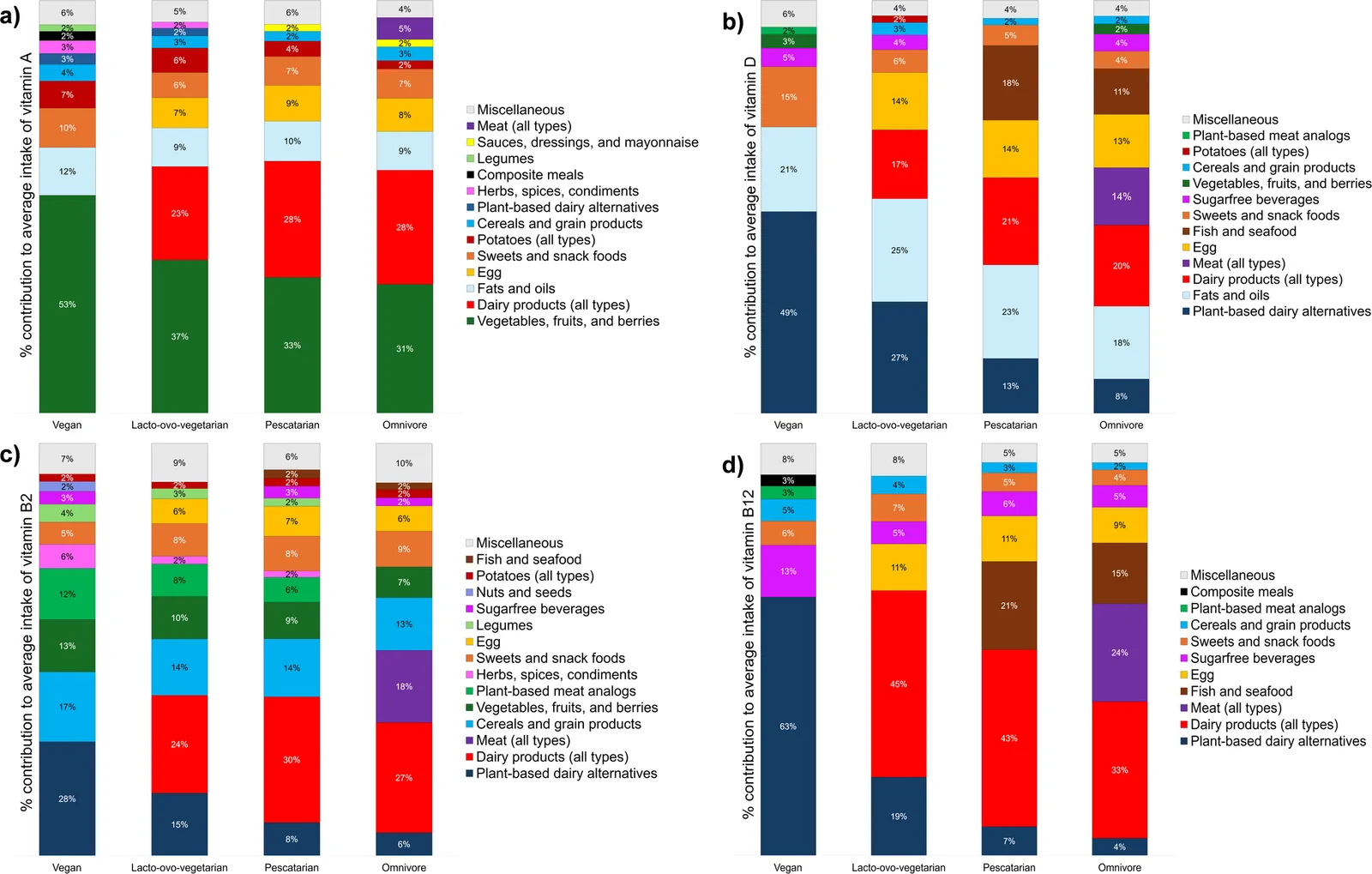
The contribution (%) of different food categories to the average intakes of **a** vitamin A, **b** vitamin D, **c** vitamin B_2_, and **d** vitamin B_12_ among youth adhering to vegan, lacto-ovo-vegetarian, pescatarian, or omnivorous diets. The category ‘miscellaneous’ includes all food categories that each contributed ≤ 1% of the average intake, and represents the sum of these categories. The category ‘herbs, spices, condiments’ includes nutritional yeast, a food rich in some B-vitamins. The category ‘composite meals’ includes composite foods (e.g., lasagne, spring rolls), where it was not possible to disaggregate them into ingredients

**Fig. 3:**
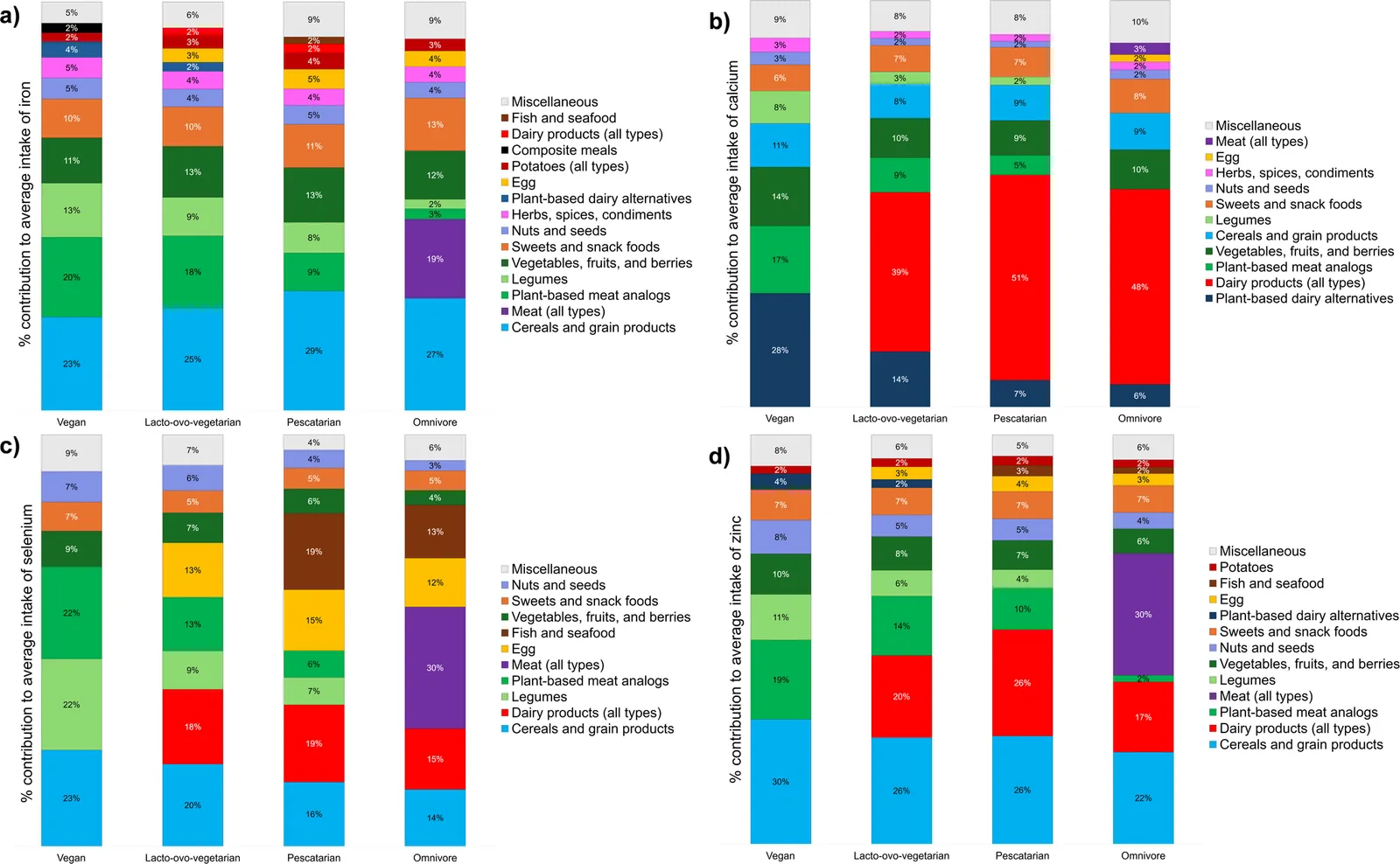
The contribution (%) of different food categories to the average intakes of **a** iron, **b** calcium, **c** selenium, and **d** zinc among youth adhering to vegan, lacto-ovo-vegetarian, pescatarian, or omnivorous diets. The category ‘miscellaneous’ includes all food categories that each contributed ≤ 1% of the average intake, and represents the sum of these categories. The category ‘composite meals’ includes composite foods (e.g., lasagne, spring rolls), where it was not possible to disaggregate them into ingredients

In Figs. 1, 2 and 3 the contribution (%) of food categories to average intakes of energy (Fig. 5a), protein (Fig. 5b), dietary fiber (Fig. 5c), SFA (Fig. 5d), vitamin A (Fig. 2a), vitamin D (Fig. 2b), B_2_ (Fig. 2c), B_12_ (Fig. 2d), iron (Fig. 3a), calcium (Fig. 3b), selenium (Fig. 3c), and zinc (Fig. 3d) are presented. Results on the contribution of food categories and dietary supplements to average intakes of vitamins (A, D, B_2_, B_12_), and minerals (iron, calcium, selenium, zinc), stratified by supplement use and dietary practice, are presented in Supplementary Tables 1–8.

#### Energy

**Fig. 4:**
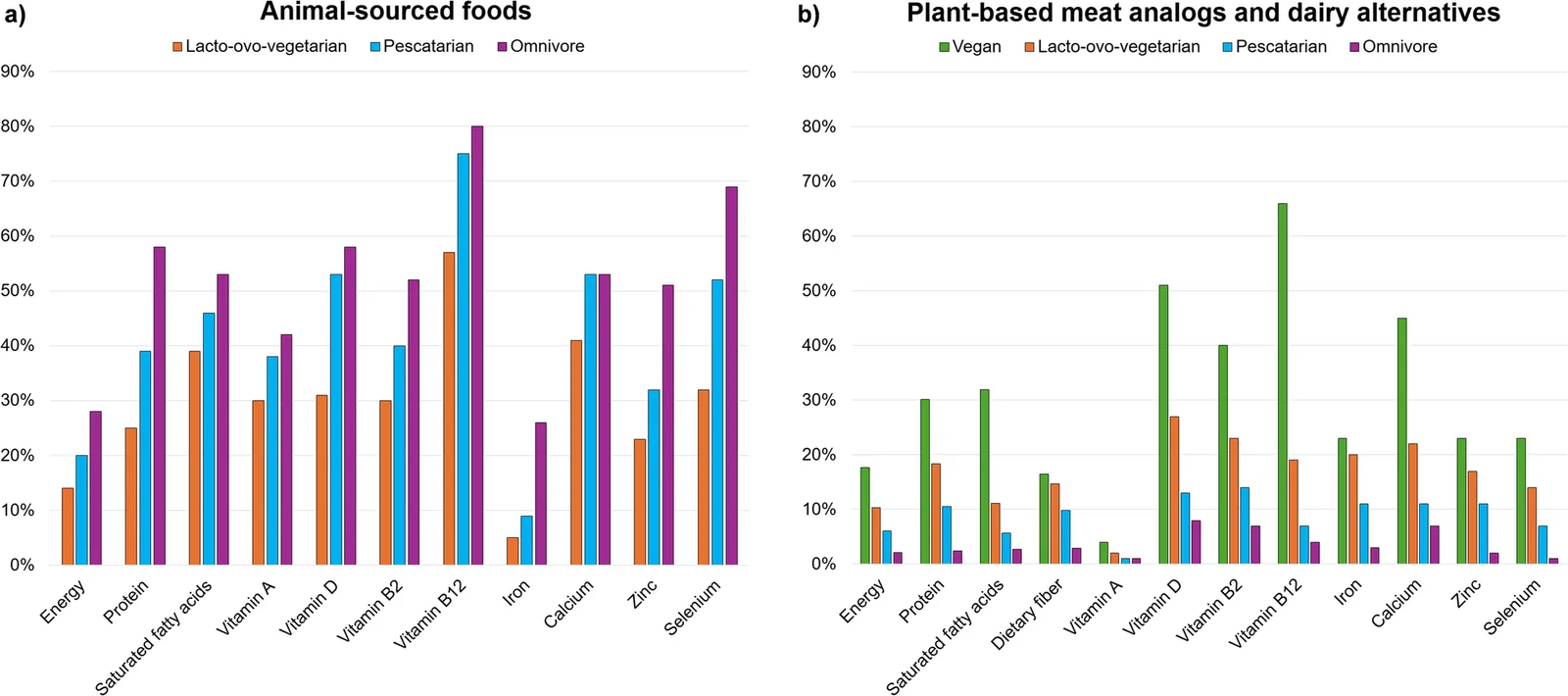
The contribution (%) of **a** animal-sourced foods (dairy products, eggs, fish and seafood, meat [all types]) and **b** plant-based meat analogs and dairy alternatives to average intakes of energy and nutrients among youth adhering to vegan, lacto-ovo-vegetarian, pescatarian, or omnivorous diets

**Fig. 5:**
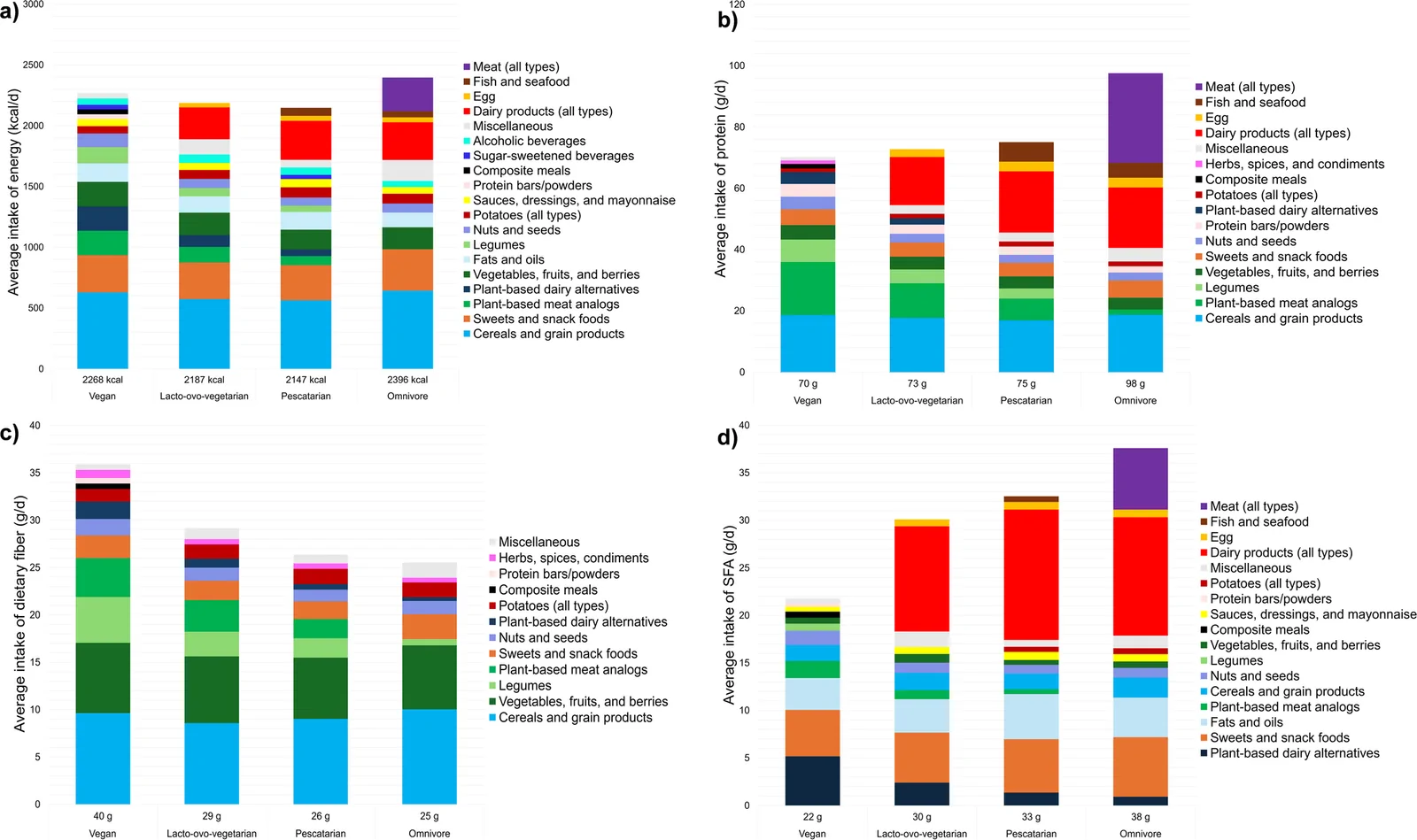
The contribution of different food categories relative to the average intake of **a** energy, **b** protein, **c** dietary fiber, and **d** saturated fatty acids (SFA) among youth adhering to vegan, lacto-ovo-vegetarian, pescatarian, or omnivorous diets. The category ‘miscellaneous’ includes all food categories that each contributed ≤ 1% of the average intake, and represents the sum of these categories. The category ‘composite meals’ includes composite foods (e.g., lasagne, spring rolls), where it was not possible to disaggregate into ingredients. Values shown below each stacked bar indicate the total average intake of the nutrient from food sources only

The contributions of several food categories to energy intake were largely similar across the dietary groups, with cereals and grain products (26–28%) and sweet and snack foods (13–14%) providing very similar proportions of energy intake. However, the contribution of plant-based dairy alternatives, plant-based meat analogs, and legumes to energy intake increased as animal-sourced food categories were excluded from the diet (highest % contribution among vegans) (Figs. 5a and 4b). Meanwhile the contribution of animal-sourced foods to energy intake increased with the greater inclusion of animal-sourced food categories in the diet; from 14% of the intake among lacto-ovo-vegetarians to 28% in omnivores (Fig 4a ). The contribution of animal-sourced foods, as well as plant-based meat analogs and dairy alternatives (combined), to energy and nutrient intakes are presented in Fig. 4 and specific values are presented in Supplementary Tables 9 and 10.

#### Protein

The food categories contributing the most to protein intake differed somewhat across dietary groups (Fig. 5b). As animal-sourced food categories were progressively excluded from the diet, protein intake was increasingly obtained from cereals and grain products, plant-based meat analogs, and legumes; vegans got 62% of their protein intake from these three food categories, while omnivores got 21% of their protein intake from them. Instead, animal-sourced foods contributed to 58% of the average protein intake among omnivores, whereof meat and dairy products together accounted for 50% (Figs. 5b ). Among lacto-ovo-vegetarians and pescatarians, protein intake was derived from a combination of dairy products (22–27%), cereals and grain products (23–24%), plant-based meat analogs (9–16%) and, among pescatarians, fish (8%).

#### Dietary fiber

Across the four dietary groups, cereals and grain products (27–39%) and vegetables, fruits, and berries (21–26%) were the top two contributors to average intakes of dietary fiber. However, legumes and plant-based meat analogs contributed more, in %, to dietary fiber intakes among vegans, lacto-ovo-vegetarians, and pescatarians than omnivores, with the highest contribution among vegans (Fig. 5c). Meanwhile, omnivores got most of their dietary fiber (65%) from cereals and grain products, and vegetables, fruits, and berries.

#### Saturated fatty acids (SFA)

The food categories contributing to SFA intakes differed somewhat across dietary groups (Fig. 5d). Among vegans, plant-based dairy alternatives were the food category contributing the most to SFA intake (24%), whereas dairy products were the largest contributor among lacto-ovo-vegetarians, pescatarians, and omnivores, providing 33–42% of the intake. Sweets and snack foods contributed similar proportions of SFA intake across all dietary groups (17–22%). Among omnivores, meat contributed 17% of SFA intake, resulting in meat and dairy products together providing approximately half of their average intake of SFA (Fig. 4a).

#### Vitamin A

Vegetables, fruits, and berries were the food category contributing the most to vitamin A intake across all four dietary groups (Fig. 2a). However, the contribution from vegetables, fruits, and berries increased progressively as more animal-sourced food categories were excluded from the diet, providing 31% of the average vitamin A intake among omnivores, and 53% among vegans. Conversely, the contribution of dairy products increased with greater inclusion of animal-sourced foods in the diet, providing 23–28% of vitamin A intake among lacto-ovo-vegetarians, pescatarians, and omnivores (Fig. 2a).

#### Vitamin D

The contribution of fortified plant-based foods to vitamin D intake increased as animal-sourced food categories were excluded from the diet (highest % contribution among vegans) (Fig. 2b). Among vegans, plant-based dairy alternatives, fats and oils, and sweets and snack foods together provided 85% of vitamin D intake, with plant-based dairy alternatives alone contributing 49% of the average intake of vitamin D. Lacto-ovo-vegetarians obtained vitamin D from a combination of fortified plant-based foods (plant-based dairy alternatives: 27%, fats and oils: 25%) and animal-sourced foods (dairy products: 17%, eggs: 14%). In contrast, animal-sourced food categories were the largest contributors of vitamin D among pescatarians and omnivores, together contributing 53 and 58% of average vitamin D intake, respectively (Fig. 4a).

#### Vitamin B_2_

Across the four dietary groups, the food categories contributing to B_2_ intake were largely similar. However, plant-based dairy alternatives and plant-based meat analogs contributed increasingly to B_2_ intake as animal-sourced food categories were excluded from the diet (highest % contribution among vegans) (Fig. 2c). Plant-based dairy alternatives were the largest contributor to B_2_ intake among vegans (28%), while dairy products were the largest contributor among lacto-ovo-vegetarians, pescatarians, and omnivores (24–30%).

#### Vitamin B_12_

The main food categories contributing to B_12_ intakes differed across the dietary groups. As animal-sourced food categories were excluded from the diet, the contribution of fortified plant-based foods to B_12_ intake increased, while the contribution of animal-sourced foods to B_12_ intake decreased (Figs. 2d and 4a). Among vegans, nearly two-thirds of their average B_12_ intake were obtained from plant-based dairy alternatives (63%), followed by sugar-free beverages (13%). In contrast, animal-sourced food categories (i.e., dairy products, eggs, fish and seafood, meat) together contributed 56% of B_12_ intake among lacto-ovo-vegetarians, 75% among pescatarians, and 81% among omnivores.

#### Iron

The food categories contributing to iron intakes were largely similar across the dietary groups, with cereals and grain products (23–29%) contributing the most iron, independent of dietary practice (Fig. 3a). The main difference was the contribution of legumes, plant-based meat analogs, and meat to iron intakes. As animal-sourced food categories were excluded from the diet, the contribution of legumes and plant-based meat analogs to iron intake increased; from 5% among omnivores to 33% among vegans. Conversely, meat was the second largest contributor to iron intake among omnivores, providing 19% of the average iron intake.

#### Calcium

Dairy products provided almost half of the calcium half among lacto-ovo-vegetarians (39%), pescatarians (51%), and omnivores (48%) (Fig. 3b). In contrast, vegans obtained calcium from several plant-based food categories, including plant-based dairy alternatives (28%), plant-based meat analogs (17%), and legumes (14%).

#### Selenium

With increasing exclusion of animal-sourced food categories from the diet, a greater proportion of selenium intake was obtained from cereals and grain products, legumes, and plant-based meat analogs (Fig. 3c). Together, these food categories contributed 67% of selenium intake among vegans, compared with 42% among lacto-ovo-vegetarians, 29% among pescatarians, and approximately 16% among omnivores. In contrast, animal-sourced foods (i.e., dairy products, eggs, fish and seafood, meat), contributed increasingly to selenium intake with greater inclusion of animal-sourced foods in the diet, providing 31% of selenium intake among lacto-ovo-vegetarians, 53% among pescatarians, and 70% among omnivores (Fig. 4a).

#### Zinc

The food categories contributing to zinc intake were relatively similar across dietary groups. Cereals and grain products were the largest or second largest contributor to zinc intake in all dietary groups, providing 22–30% of the average intake. With increasing exclusion of animal-sourced food categories from the diet, a greater proportion of zinc intake was obtained from plant-based meat analogs and legumes, which together contributed 30% of average zinc intake among vegans compared with 2% among omnivores (Fig. 3d). Conversely, the contribution of animal-sourced foods to zinc intake increased with greater inclusion of animal-sourced food categories in the diet; dairy products contributed 17–26% of the average zinc intake among the non-vegan dietary group and meat additionally provided 30% of average zinc intake among omnivores.

### Average intakes of energy and nutrient intakes

**Fig. 6:**
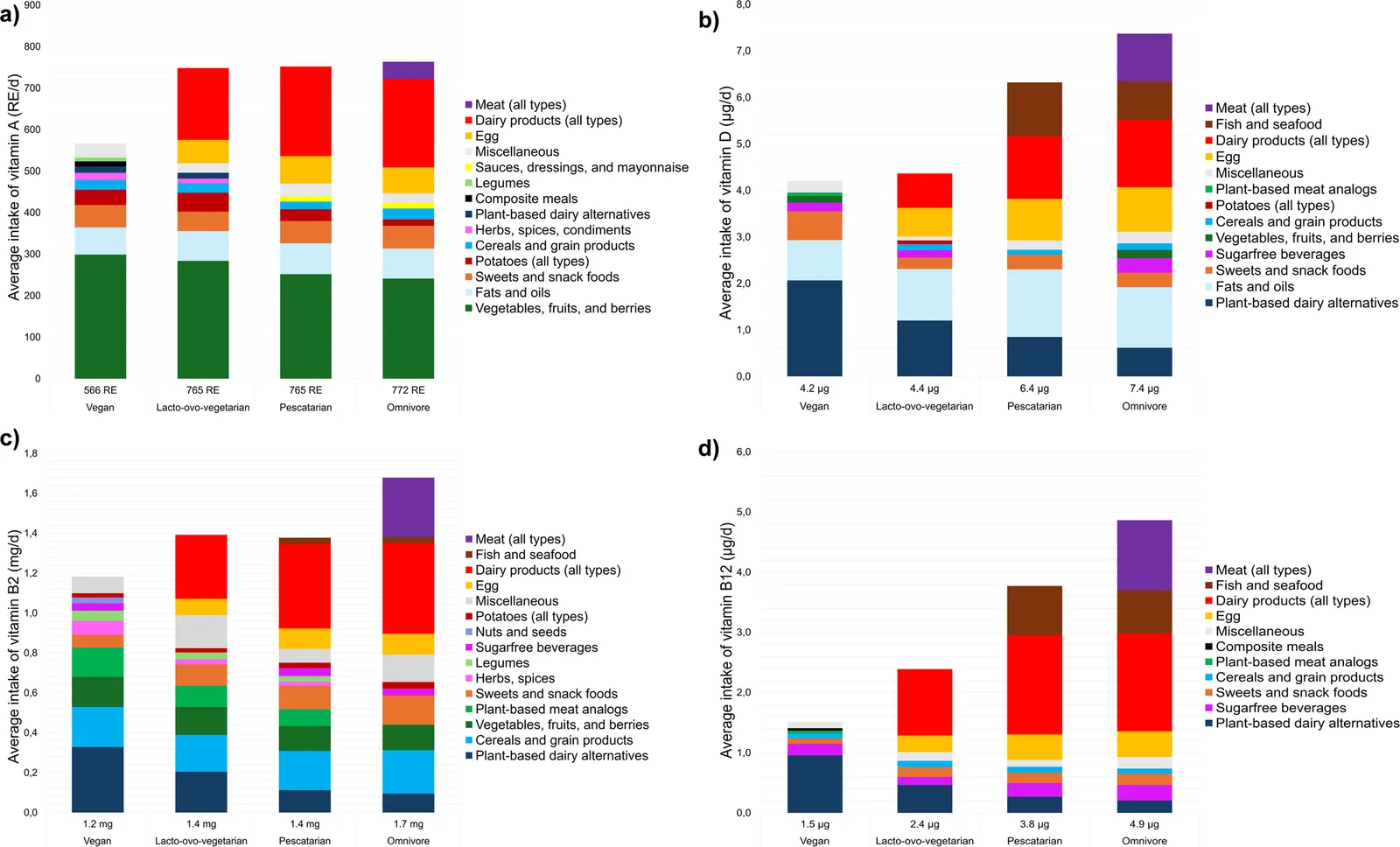
The contribution of different food categories relative to the average intake of **a** vitamin A, **b** vitamin D, **c** vitamin B_2_, and **d** vitamin B_12_ among youth adhering to vegan, lacto-ovo-vegetarian, pescatarian, or omnivorous diets. The category ‘miscellaneous’ includes all food categories that each contributed ≤ 1% of the average intake, and represents the sum of these categories. The category ‘composite meals’ includes composite foods (e.g., lasagne, spring rolls), where it was not possible to disaggregate into ingredients. Values shown below each stacked bar indicate the total average intake of the nutrient from food sources only

**Fig. 7:**
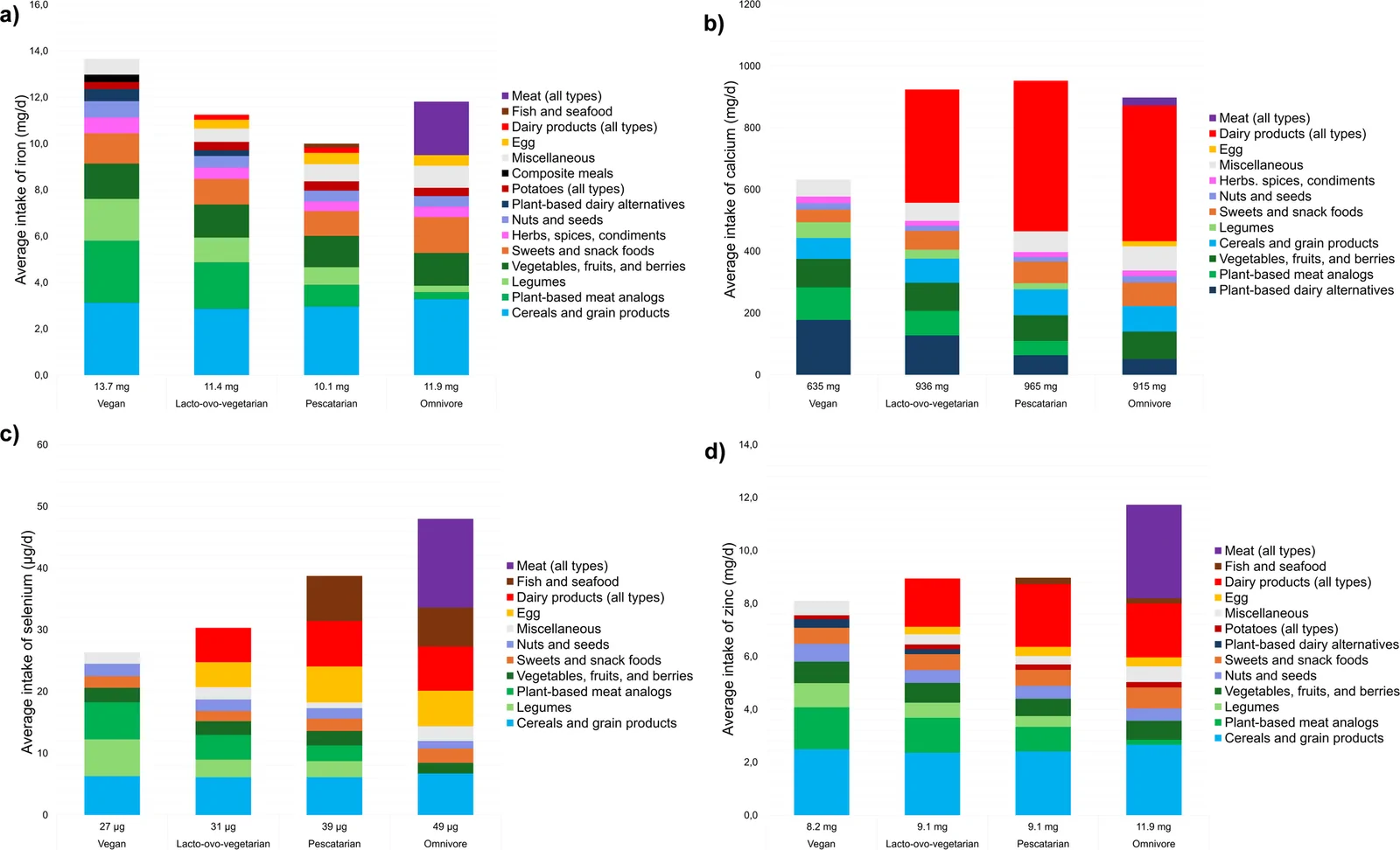
The contribution of different food categories relative to the average intake of **a** iron, **b** calcium, **c** selenium, and **d** zinc among youth adhering to vegan, lacto-ovo-vegetarian, pescatarian, or omnivorous diets. The category ‘miscellaneous’ includes all food categories that each contributed ≤ 1% of the average intake, and represents the sum of these categories. The category ‘composite meals’ includes composite foods (e.g., lasagne, spring rolls), where it was not possible to disaggregate into ingredients. Values shown below each stacked bar indicate the total average intake of the nutrient from food sources only

To contextualise the contributions (%) of food categories to average intakes shown in Figs. 1, 2 and 3, the contributions, relative to average energy and nutrient intakes, are presented in Figs. 5, 6 and 7.

The average intake of energy was generally similar across the dietary groups, but omnivores had the highest energy intake and pescatarians the lowest. For protein, SFA, vitamins A, D, B_2_, and B_12_, selenium, and zinc, average intakes generally decreased as animal-sourced food categories were progressively excluded from the diet, whereas dietary fiber intake increased. Vegans had the lowest average intakes of these nutrients, and the highest dietary fiber intake, while omnivores generally showed the opposite pattern (Figs. 5, 6 and 7). Lacto-ovo-vegetarians and pescatarians had lower iron intakes than among both vegans and omnivores (Fig. 7a), while vegans had the lowest and pescatarians the highest calcium intake (Fig. 7b).

## Discussion

### Main key findings

This study is the first since the late 1990’s to assess the contribution of food categories to energy and nutrient intakes among youth adhering to plant-based (vegan, lacto-ovo-vegetarian, pescatarian) or omnivorous diets in Sweden. Our findings indicate that nutrients which are typically provided by animal-sourced foods in omnivorous diets are instead obtained through a combination of plant-based foods when animal-sourced food categories are limited or excluded from the diet. In particular, the contribution of plant-based meat analogs, plant-based dairy alternatives, and legumes to energy and nutrient intakes increased when more animal-sourced food categories were excluded from the diet. Furthermore, the prominent contribution of fortified plant-based dairy alternatives to intakes of vitamins D, B_12_ and calcium in plant-based diets, especially vegan diets, highlights the role of food fortification in supporting nutrient adequacy in plant-based dietary practices.

### The role of plant-based meat analogs to nutrient intakes in plant-based diets

As more animal-sourced food categories were excluded from the diet, plant-based meat analogs, legumes, and cereals and grain products became increasingly important dietary sources of protein, as well as several micronutrients, including iron, selenium, and zinc. This shift is broadly consistent with the Nordic Nutrition Recommendations (NNR) 2023, which recommend that decreased meat consumption ought to be replaced by increased consumption of plant-based foods, such as legumes, as well as fish from sustainably managed stocks [2]. However, notably, in this present study, plant-based meat analogs were the second and third largest contributor to protein intake among vegans and lacto-ovo-vegetarians, providing 16–25% of the average protein intake. This contrasts with findings from the German *VeChi Youth* study, in which cereals and grain products accounted for a larger proportion of protein intake (36–39%) among young vegans and lacto-ovo-vegetarians, whereas meat analogs contributed less than 12% of total protein intake 20]. The observed difference may reflect differences in the food environment between Sweden and Germany. For example, a report from 2022 showed that Sweden was among the leading European countries in per capita consumption of plant-based meat analogs [30], and almost all (≥ 95%) participants adhering to plant-based diets (vegan, lacto-ovo-vegetarian, pescatarian) in the present study reported consuming plant-based meat analogs (Table 2). The widespread availability and consumption of these products may therefore explain the contribution that plant-based meat analogs had to the energy and nutrient intakes of youth adhering to plant-based diets in the present study. However, differences in the age of the study populations (6–18 years in the *VeChi Youth* study versus 16–24 years in the present study) may also partly explain the contrasting findings regarding plant-based meat analogs.

Plant-based meat analogs offer both nutritional benefits and limitations. Compared with the meat products they are intended to replace, many contain less SFA, provide dietary fiber, and some provide comparable amounts of protein [14]. However, some types of plant-based meat analogs also contain relatively high amounts of sodium, and few are naturally rich in, or fortified with nutrients commonly obtained from meat and fish, such as vitamins D, B_2_, and B_12_, as well as selenium and iodine [14]. Furthermore, although many plant-based meat analogs are a source of iron, research suggests that the bioavailability of this iron is often low, partly due to their phytate content [15]. Nevertheless, plant-based meat analogs are a heterogeneous product category, and their nutritional content can vary considerably depending on ingredients and level of fortification. Consequently, the nutritional impact of replacing meat with plant-based meat analogs is likely to depend on the specific products chosen and the overall composition of the diet. Given the observed consumption of plant-based meat analogs in contemporary plant-based diets, further research is warranted to investigate the long-term health and nutritional effects of substituting meat with these products in a Swedish context.

### The role of fortified plant-based dairy alternatives to nutrient intakes in plant-based diets

#### Protein

Compared with dairy products in the non-vegan dietary groups, plant-based dairy alternatives contributed relatively little (5%) to protein intake among vegans. This likely reflect the low protein content of many plant-based diary alternatives [16]. A previous Swedish study reported that only soy-based alternatives have a protein content (per 100 gram) comparable to products based on cow’s milk, whereas oat-, almond-, rice-, coconut-, potato- and many pea-based alternatives typically contain less protein [16]. Nevertheless, average intakes of protein were similar among vegans, lacto-ovo-vegetarians, and pescatarians in the present study (Fig. 5b). Instead, vegans obtained protein from other sources, including cereals and grain products, meat analogs, and legumes (Fig. 5b and 5b). This highlights the importance of consuming foods from a variety of food categories to support meeting nutrient recommendations.

#### Vitamin D and B_12_

Plant-based dairy alternatives (fortified) were main contributors to intakes of vitamins D and B_12_ among vegans, as well as lacto-ovo-vegetarians, in the present study. This is unsurprising given that plant-based foods contain only trace amounts of B_12_ unless fortified [9, 31], and few naturally provide vitamin D, except for some mushrooms which have been exposed to ultraviolet light [32]. In Sweden, specific types of dairy products (cow’s milk and yoghurt), including the plant-based dairy alternatives, and margarines are mandatorily fortified with vitamin D [19], while many plant-based milk and yoghurt alternatives are commonly additionally fortified with B_12_, B_2_, and calcium [16]. Plant-based milk and yoghurt alternatives typically contain 0.75–1.10 µg vitamin D and 0.38–0.60 µg B_12_ per 100 gram; similar to the content of these nutrients in cow’s milk and yoghurt in Sweden [16]. The widespread fortification of these products contributes to more dietary sources of vitamins D and B_12_ in both vegan and non-vegan diets.

The prominent role of fortified foods observed in the present study likely reflects both Swedish fortification policies and the increased availability of fortified plant-based products on the food market. Compared with young Swedish vegans in the late 1990’s, who obtained almost all B_12_ from supplements and approximately half of their vitamin D intake from fortified margarine [21], vegans in the present study obtained these nutrients from a broader range of fortified foods. This included plant-based dairy alternatives, fats and oils, sugar-free beverages, and sweets and snack foods. Similar findings have been reported in Germany and Finland where fortified foods were also main contributors to B_12_ intake among vegans [20, 22]. However, the specific food sources differed between the studies; adults and children following vegan diets in Finland obtained most (83–91%) of their B_12_ intake from fortified plant-based dairy alternatives [22], whereas children and adolescents following vegan diets in Germany obtained most of their B_12_ intake from sweetened foods (38.8%), followed by plant-based dairy alternatives (30.4%) [20]. Notably, vegans in the present study obtained a considerably larger proportion of their B_12_ intake from plant-based dairy alternatives (63% vs. 30.4%) and a smaller proportion from sweetened foods (6% vs. 38.8%) than vegan children/adolescents in Germany [20]. Compared with Sweden, the availability of fortified plant-based drinks is considerably lower in Germany, where approximately only 20% of plant-based dairy alternatives on the market are fortified with at least one nutrient [33]. Thus, availability of fortified foods plays a role in which food sources provide the most nutrients, especially so for vegan diets.

#### Calcium

In contrast to the non-vegan dietary groups, where dairy products provided 39–51% of calcium intake, vegans in this present study obtained calcium from a broader range of food categories, including plant-based dairy alternatives (fortified), plant-based meat analogs (including tofu), legumes, cereals and grain products, and vegetables, fruits, and berries. Similar food sources have been reported among vegan children and adolescents in Germany, and adults and children (families) consuming vegan diets in Finland, indicating that calcium is typically obtained from several food categories in vegan diets rather than being obtained primarily from one food category [20, 22]. Nevertheless, similar to findings from Germany and Finland, fortified plant-based dairy alternatives were a main source of calcium among vegans in the present study [20, 22]. However, the contribution of plant-based dairy alternatives to calcium differs across studies, with this food category providing 28% of the calcium intake among vegans in the present study compared with 19% among vegan children and adolescents in Germany, and 46–73% among vegan children and adults in Finland [20, 22]. This highlights the role of (fortified) plant-based dairy alternatives in supporting calcium intakes when dairy products are excluded from the diet. Nevertheless, despite the contribution of fortified foods, intakes of several nutrients including calcium, remained lower among vegans than non-vegans in the present study (Fig. 7b). This suggests that fortified foods can be an important replacement for animal-sourced foods but may not fully compensate for the exclusion of animal-sourced foods from the diet, especially not at youths’ current consumption levels of these products. Consistent with this, the Swedish Food Agency recommends the regular consumption of fortified plant-based drinks and yoghurt alternatives, whole grains, legumes, nuts and seeds, vegetables (particularly dark leafy greens) and fruitsas part of a nutritionally adequate vegan or lacto-ovo-vegetarian diet [11, 34].

### The role of animal-sourced foods to nutrient intakes

Animal-sourced foods were main contributors to several nutrient intakes among omnivores, which is in line with previous studies among the general Swedish population [26, 35]. In the present study, animal-sourced food categories accounted for more than half (> 50%) of the average intakes of protein, vitamins D, B_2_ and B_12_, as well as calcium, selenium, and zinc among omnivores (Fig. 4a), which closely aligns with the contributions from these animal-sourced food categories to nutrient intakes previously reported among Swedish adolescents [26]. Animal-sourced foods were also main sources of some nutrients among lacto-ovo-vegetarians and pescatarians in the present study, particularly for protein, vitamin D, B_12_, B_2_, calcium, selenium, and zinc (Fig. 4a). Notably, average intakes of protein, SFA, vitamins A, D, B_2_ and B_12_, and calcium, selenium and zinc generally decreased as animal-sourced foods were progressively excluded from the diet, with omnivores generally having the highest average intakes and vegans the lowest (Figs. 5, 6 and 7). Similar patterns of decreasing intakes of some nutrients have been reported in previous studies among adults and/or children and adolescents adhering to plant-based (vegan, lacto-ovo-vegetarian, semi-vegetarian) compared to omnivorous diets [6, 7]. These consistent findings underline the role of animal-sourced foods as sources of some key nutrients. At the same time, animal-sourced foods, in particular dairy products and meat, contributed between 39 and 53% of the average SFA intake among lacto-ovo-vegetarians, pescatarians, and omnivores in the present study (Fig. 4a). The average intake of SFA exceeds dietary recommendations of 10 E% in the Nordic population, and the NNR 2023 recommend replacing some SFA intake with polyunsaturated fatty acids, whole grains, and other fiber-rich plant foods, which can improve blood lipid profiles and may reduce the risk of coronary heart disease later in life [2]. Thus, the food categories that contributed most to several key nutrients of public health importance, e.g., vitamin D, B_12_, iron, and calcium, were also main contributors to SFA intake, highlighting the nutritional trade-offs associated with animal-sourced foods. Strategies such as choosing low-fat dairy products (or fortified plant-based dairy alternatives) and replacing some red meat with fish, or plant-sources of protein, as recommended by the NNR 2023, can help reduce SFA intake [2]. However, it remains important to ensure that intakes of key micronutrients meet dietary recommendations when reducing intakes of animal-sourced foods.

### Main food sources of energy in plant-based and omnivorous diets

Despite differences in dietary practice and the number of animal-sourced food categories included in the diet, the main contributors to energy intake were broadly similar across dietary groups. Cereals and grain products contributed the most energy (26–28%), followed by sweets and snack foods (13–14%) or dairy products (12–15%). This is consistent with findings among adolescents in the general Swedish population, where cereals and grain products, and sweets and snack foods were found to contribute approximately 20 and 13% of total energy intake, respectively [26]. Similarly, studies among children and adolescents in Germany and families in Finland, reported that cereals and grain products, followed by sweets and snack foods, or dairy/plant-based dairy alternatives were the largest contributors to energy intake irrespective of vegan, lacto-ovo-vegetarian, or omnivorous diet [20, 22]. Given that cereals and grains are a main source of energy, the type of grain products consumed is relevant for nutrient intakes. Whole grains provide more dietary fiber, vitamins, and minerals than refined grains andhave been associated with a lower risk of cardiovascular disease, type-2 diabetes, colorectal cancer, and premature mortality [36]. However, we previously reported that adherence to the food-based dietary guideline of ≥ 90 g whole grains/day by the NNR 2023 was low among youth in the present study [24]. Thus, suggesting that most of the energy intake from cereals and grains may come from refined grain products. Supporting youth to increase their intakes of whole grain foods, in replacement of refined grains, could represent an effective strategy for improving micronutrient intakes and promoting long-term health, irrespective of plant-based or omnivorous diet.

Sweets and snack foods were the second or third largest contributor to energy intake across all dietary groups in the present study, contributing to 13–14% of the average energy intake. This closely resembles the contribution reported among the general Swedish population, where sugar-sweetened foods and beverages as well as snack foods were found to contribute approximately 15–17% of total energy intake [26, 35]. Similar findings have also been reported among German children and adolescents adhering to vegan, lacto-ovo-vegetarian, or omnivorous diets, where sweets and snack foods contributed 12–16% of energy intake [20], and among Swedish adolescents adhering to vegan and omnivorous diets in the late 1990’s, these foods contributed 16–22% of the energy intake [21]. Together, these findings suggest that consumption of sweets and snack foods is common among youth, and are a main source of energy, irrespective of plant-based or omnivorous diet.

Notably, sweets and snack foods, and beverages (both sugar-sweetened and sugar-free alternatives) contributed to intakes of several micronutrients among vegans in the present study. This is largely due to the fortification of these foods or the ingredients used in their production (e.g., fortified margarine in baked goods). In Sweden, voluntary fortification of foods with vitamins and minerals is permitted in accordance with EU Regulation No. 1925/2006. Although these products contributed somewhat to youths’ nutrient intakes, sweets, snack foods, and sweetened caffeinated beverages are generally not considered optimal sources of key nutrients. For example, sweets and snack foods simultaneously contribute to intakes of free sugars and SFA, and a high consumption of sugar-rich foods has been associated with a lower likelihood of meeting nutrient recommendations of key nutrients among Swedish adolescents [37]. From a public health perspective, youth should be encouraged to replace some of their intakes of sweets and snack foods, and sugar-sweetened beverages with more nutrient dense foods, such as whole grains, vegetables, fruits and berries, nuts and seeds, legumes, low-fat dairy products, and/or fortified plant-based dairy alternatives. This may help support youth achieve higher intakes of key micronutrients.

### Implications for dietary guidance for plant-based diets

A global shift towards diets based on larger amounts of plant-based foods is encouraged because of their potential health and environmental benefits [1, 2]. However, to support individuals consume well-planned and nutritionally adequate plant-based diets, it is important that dietary guidance is available on how to obtain adequate intakes of nutrients from plant-based foods. Current dietary guidance for vegan and lacto-ovo-vegetarian diets commonly advises consuming fortified foods and, for some nutrients such as vitamin B12 and D, supplementing with these nutrients, especially when adhering to a vegan diet [9-11, 13]. However, guidance is often less explicit regarding the quantities and combinations of plant-based foods needed to replace animal-sourced foods and support intakes of nutrients that meet dietary recommendations. In the present study we found that average intakes of several nutrients including vitamin D, B12, and calcium, remained lower among vegans than non-vegans, despite consumption of fortified foods. Furthermore, we previously reported that across dietary groups, youth in the present study were at risk of inadequate intake of vitamin D and selenium, which remained when assessing intakes from both foods and supplements [23]. Meanwhile, vegans and lacto-ovo-vegetarians were additionally at risk of inadequate intake of other nutrients [23]. More vegans and lacto-ovo-vegetarians reported supplement use than omnivores in the present study (Table 1 and Supplementary Tables 1-8) [23]. This suggests that fortified foods, dietary supplements, and other nutrient-rich foods may not always be consumed in sufficient amounts among young vegans and lacto-ovo-vegetarians to fully replace the nutrients provided by meat and fish, as well as eggs and dairy (among vegans). Dietary guidance for vegan and lacto-ovo-vegetarian diets should therefore provide practical information on how animal-sourced foods can be nutritionally replaced through appropriate food choices, fortified foods, and, when necessary, supplements. As foods contribute more than individual nutrients, supporting adequate nutrient intakes through foods remains important. Youth may therefore benefit from strengthening their food-related knowledge and skills needed to select, plan, and prepare nutritionally adequate plant-based meals. Additionally, having sufficient knowledge of appropriate supplement use, when necessary, to secure adequate nutrient intakes [9, 11]. 

### Strengths and limitations

A strength of this study is that the majority of youth completed all of the four 24HDRs, which provided detailed dietary intake data. Although, the dietary intake data is self-reported, and therefore there is inevitably a risk for under- and overreporting. However, we previously reported that in this study there was a very low number of youth who were classified as having underreported their intake (7%) [24]. Furthermore, a strength of the study is the thorough examination of almost all nutrients in the human diet, and from different food categories and supplements. Furthermore, the dietary groups were similar in size and demographics, enabling fair comparisons between the dietary groups. Nevertheless, a limitation of the study is that the sample consisted of mostly females who had a high education level and lived in the city, which impacts the generalizability of our results. Yet, these demographics mostly align with the study samples in similar cross-sectional studies conducted in Western countries [5, 20], and also the demographic of those reporting consuming plant-based diets in Sweden [4] as well as other European countries [3, 5].

This study also has limitations. Consumer status was determined from two to four 24HDRs collected over 2–7 weeks and reflects reported intake during the assessment period. Consequently, some sporadic consumers may have been misclassified as non-consumers. Another limitation is that for some foods, including novel plant-based foods and ready-made meals, there was no nutritional information for these foods in the Food Composition Databases. This is an issue which has been noted in other similar studies [5, 38]. How missing food items were handled in the current study has been described in detail elsewhere [23]. Briefly, either a similar item from the Swedish Food Composition Database was selected, nutritional information for the product was entered into the dietary assessment program, or standardized recipes were created based on the ingredient list and matched to the nutritional content of the product, or using popular Swedish online recipes (see method section). For some food items, such as vegan baked goods (pastries, buns, cookies) and potato dishes, standardized recipes (using online recipes) were created as replacement food items. Fortified products (i.e., plant-based dairy alternatives and margarine) were used in these recipes. However, mandatory fortification of vitamin D in cow’s milk and yoghurt and plant-based alternatives, and fortification of vitamins A and D in margarine only applies to products intended for final consumers or mass catering [19]. It was not always possible to discern if the food item was home-made or storebought. Consequently, contributions of sweets and snack foods to intakes of vitamins A, D, and B_12_ may have been slightly overestimated if these food items were primarily premade or store-bought.

## Conclusion

As animal-sourced foods were progressively excluded from youths’ diet, energy and nutrient intakes were increasingly obtained from cereals and grain products, plant-based meat analogs, plant-based dairy alternatives, and legumes. Fortified plant-based dairy alternatives were main contributors to intakes of vitamin D, B_12_, and calcium among vegans, whereas animal-sourced foods remained the main sources of these nutrients among lacto-ovo-vegetarians, pescatarians, and omnivores. Replacing animal-sourced foods with plant-based alternatives led to increasingly higher intakes of dietary fiber and lower intakes of SFA. Nevertheless, despite several food categories contributing to nutrient intakes, average intakes of several nutrients (protein, vitamins A, D, B_2_, B_12_, calcium, selenium, and zinc) generally decreased as more animal-sourced food categories were excluded from the diet, with vegans having the lowest average intakes of these nutrients, from food sources only. These findings suggest that particular attention should be given to the food categories contributing to key nutrient intakes (i.e., vitamin D, B_12_, calcium [vegans only] and selenium) when planning vegan and lacto-ovo-vegetarian diets, and promoting intakes of nutrient-rich plant foods, regular consumption of fortified products, and, where necessary, appropriate supplementation may be needed.

## Supplementary Information

Below is the link to the electronic supplementary material.

Supplementary Material 1

## Data Availability

The data generated and analyzed in the current study is not publicly available. However, data can be made available on reasonable request. Requests can be sent to the corresponding author.

## Abbreviations

AR: Average requirements
B_12_: Vitamin B_12_
B_2_: Vitamin B_2_
NNR2023: Nordic Nutrition Recommendations 2023
SFA: Saturated fatty acids
SD: Standard deviations
24HDR: 24-hour dietary recalls
