# Supplementary material for: Food categories contributions to energy and nutrient intakes among youth adhering to vegan, lacto-ovo-vegetarian, pescatarian or omnivorous diets
Source: Eur J Nutr. 2026 Oct 31;65(8):289. doi: 10.1007/s00394-026-04128-y (PMC13723639; doi:10.1007/s00394-026-04128-y)
Supplement: Supplementary file 1 [file 394_2026_4128_MOESM1_ESM.pdf]

Food categories contributions to energy and nutrient intakes among youth adhering to vegan, lacto-ovo-vegetarian, pescatarian, or omnivorous diets

Authors: Isabelle Mulkerrins[1], Anine Christine Medin[2], Synne Groufh-Jacobsen[3], Claire Margerison[4] and Christel Larsson[1].

[1] Department of Food and Nutrition, and Sport Science, Faculty of Education, University of Gothenburg, PO Box 300, SE-405 30 Gothenburg, Sweden.

[2] Department of Nutrition and Public Health, Faculty of Health and Sport Sciences, University of Agder, Universitetsveien 25, 4630 Kristiansand, Norway.

[3] Department of Health Sciences, Oslo New University College, Oslo, Norway.

[4] Institute for Physical Activity and Nutrition, Deakin University, Geelong, Australia.

Tables

Supplementary Table 1 Contribution (%) from food categories and dietary supplements to average intakes of vitamin A (RE/d) among youth, stratified by supplement use and dietary practice (vegan, lacto-ovo-vegetarian, pescatarian, omnivore)

Supplementary Table 2 Contribution (%) from food categories and dietary supplements to average intakes of vitamin D (µg/d) among youth, stratified by supplement use and dietary practice (vegan, lacto-ovo-vegetarian, pescatarian, omnivore)

Supplementary Table 3 Contribution (%) from food categories and dietary supplements to average intakes of vitamin B_2 (mg/d) among youth, stratified by supplement use and dietary practice (vegan, lacto-ovo-vegetarian, pescatarian, omnivore)

Supplementary Table 4 Contribution (%) from food categories and dietary supplements to average intakes of vitamin B_{12} (µg/d) among youth, stratified by supplement use and dietary practice (vegan, lacto-ovo-vegetarian, pescatarian, omnivore)

Supplementary Table 5 Contribution (%) from food categories and dietary supplements to average intakes of iron (mg/d) among youth, stratified by supplement use and dietary practice (vegan, lacto-ovo-vegetarian, pescatarian, omnivore)

Supplementary Table 6 Contribution (%) from food categories and dietary supplements to average intakes of calcium (mg/d) among youth, stratified by supplement use and dietary practice (vegan, lacto-ovo-vegetarian, pescatarian, omnivore)

Supplementary Table 7 Contribution (%) from food categories and dietary supplements to average intakes of selenium (µg/d) among youth, stratified by supplement use and dietary practice (vegan, lacto-ovo-vegetarian, pescatarian, omnivore)

Supplementary Table 8 Contribution (%) from food categories and dietary supplements to average intakes of zinc (mg/d) among youth, stratified by supplement use and dietary practice (vegan, lacto-ovo-vegetarian, pescatarian, omnivore)

Supplementary Table 9 Contribution (%) from animal-sourced foods (dairy products, eggs, fish and seafood, meat [all types]) to average intakes of energy, protein, saturated fatty acids, and micronutrients among vegans, lacto-ovo-vegetarians, pescatarians, and omnivores aged 16-24 years old

Supplementary Table 10 Contribution (%) from plant-based meat analogs and plant-based dairy alternatives to average intakes of energy, protein, saturated fatty acids, and micronutrients among vegans, lacto-ovo-vegetarians, pescatarians, and omnivores aged 16-24 years old

Figures

Supplementary Fig 1 The contribution (%) of different food categories to the average intakes of a) carbohydrates, b) total fat, c) monounsaturated fatty acids (MUFA), and d) polyunsaturated fatty acids (PUFA) among youth adhering to vegan, lacto-ovo-vegetarian, pescatarian, or omnivorous diets.

Supplementary Fig 2 The contribution (%) of different food categories to the average intakes of a) omega-3 fatty acids, b) omega-6 fatty acids and c) free sugars among youth adhering to vegan, lacto-ovo-vegetarian, pescatarian, or omnivorous diets.

Supplementary Fig 3 The contribution (%) of different food categories to the average intakes of a) vitamin B_1, b) vitamin B_6, c) vitamin C, and d) niacin among youth adhering to vegan, lacto-ovo-vegetarian, pescatarian, or omnivorous diets.

Supplementary Fig 4 The contribution (%) of different food categories to the average intakes of a) vitamin K, b) vitamin E, c) folate, and d) magnesium among youth adhering to vegan, lacto-ovo-vegetarian, pescatarian, or omnivorous diets.

Supplementary Fig 5 The contribution (%) of different food categories to the average intakes of a) potassium and b) phosphorous among youth adhering to vegan, lacto-ovo-vegetarian, pescatarian, or omnivorous diets

Supplementary Fig 6 The contribution of different food categories relative to the average intake of a) carbohydrates, b) total fat, c) monounsaturated fatty acids (MUFA), and d) polyunsaturated fatty acids (PUFA) among youth adhering to vegan, lacto-ovo-vegetarian, pescatarian, or omnivorous diets.

Supplementary Fig 7 The contribution of different food categories relative to the average intake of a) omega-3 fatty acids, b) omega-6 fatty acids, and c) free sugars among youth adhering to vegan, lacto-ovo-vegetarian, pescatarian, or omnivorous diets.

Supplementary Fig 8 The contribution of different food categories relative to the average intake of a) vitamin B_1, b) vitamin B_6, c) vitamin C, and d) niacin among youth adhering to vegan, lacto-ovo-vegetarian, pescatarian, or omnivorous diets.

Supplementary Fig 9 The contribution of different food categories relative to the average intake of a) vitamin K, b) vitamin E, c) folate, and d) magnesium among youth adhering to vegan, lacto-ovo-vegetarian, pescatarian, or omnivorous diets.

Supplementary Fig 10 The contribution of different food categories relative to the average intake of a) potassium and b) phosphorous among youth adhering to vegan, lacto-ovo-vegetarian, pescatarian, or omnivorous diets.

Supplementary Table 1 Contribution (%) from food categories and dietary supplements to average intakes of vitamin A (RE/d) among youth, stratified by supplement use and dietary practice (vegan, lacto-ovo-vegetarian, pescatarian, omnivore)

	Supplement user*				Non-supplement user			
	Veg	LOV	Pesc	Omni	Veg	LOV	Pesc	Omni
Number of participants (n)	n=11	n=4	n=5	n=3	n=49	n=55	n=50	n=58
Alcoholic beverages	0%	0%	0%	0%	0%	0%	0%	0%
Legumes	1%	1%	0%	0%	2%	0%	1%	0%
Fish and seafood	0%	0%	1%	0%	0%	0%	1%	1%
Fruit and vegetable juice	1%	0%	6%	0%	1%	1%	1%	0%
Vegetables, fruits, and berries	36%	39%	25%	15%	53%	36%	33%	32%
Coffee and tea	0%	0%	0%	0%	0%	0%	0%	0%
Protein bars/powders	0%	0%	0%	0%	1%	0%	0%	0%
Meat (all types)	0%	0%	0%	3%	0%	0%	0%	5%
Fats and oils	3%	4%	7%	9%	12%	10%	10%	9%
Dairy products (all types)	0%	12%	20%	21%	0%	23%	28%	28%
Nuts and seeds	0%	0%	0%	0%	0%	0%	0%	0%
Potatoes (all types)	6%	0%	4%	12%	6%	6%	4%	1%
Composite meals	4%	0%	0%	0%	1%	1%	0%	0%
Sugar-sweetened beverages	0%	0%	0%	0%	2%	0%	0%	0%
Cereals and grain products	2%	2%	2%	8%	4%	3%	2%	3%
Jams, marmalades, and compotes	0%	0%	0%	0%	0%	0%	0%	0%
Sauces, dressings, and mayonnaise	1%	1%	1%	0%	1%	1%	2%	2%
Sweets and snack foods	5%	2%	4%	5%	9%	6%	7%	7%
Plant-based dairy alternatives	2%	0%	0%	3%	2%	2%	1%	1%
Plant-based meat analogs	1%	0%	0%	0%	1%	0%	0%	0%
Egg	0%	1%	6%	5%	0%	8%	9%	8%
Herbs, spices, condiments	1%	1%	1%	1%	4%	2%	1%	1%
Dietary supplements	38%	35%	21%	17%	0%	0%	0%	0%
Average intake of vitamin A (RE/d)	896	791	888	1148	561	782	773	762

Veg = vegan, LOV = lacto-ovo-vegetarian, Pesc = pescatarian, Omni = omnivore

*Supplement user was defined as having reported any type of vitamin/mineral supplement containing vitamin A in at least one of their 24-hour dietary recalls. Data is shown for descriptive purposes; no statistical tests of differences were conducted. Intakes were not normally distributed. Average intakes from 24-hour dietary recalls (between 2-4 per participant).

Supplementary Table 2 Contribution (%) from food categories and dietary supplements to average intakes of vitamin D (µg/d) among youth, stratified by supplement use and dietary practice (vegan, lacto-ovo-vegetarian, pescatarian, omnivore)

	**Supplement user***				**Non-supplement user**			
	Veg	LOV	Pesc	Omni	Veg	LOV	Pesc	Omni
Number of participants (n)	n=33	n=19	n=12	n=8	n=27	n=40	n=43	n=53
Alcoholic beverages	0%	0%	0%	0%	0%	0%	0%	0%
Legumes	0%	0%	0%	0%	0%	0%	0%	0%
Fish and seafood	0%	0%	9%	10%	0%	1%	18%	10%
Fruit and vegetable juice	0%	0%	0%	0%	0%	0%	0%	0%
Vegetables, fruits, and berries	1%	0%	0%	0%	4%	1%	0%	3%
Coffee and tea	0%	0%	0%	0%	0%	0%	0%	0%
Protein bars/powders	1%	1%	0%	0%	1%	0%	0%	0%
Meat (all types)	0%	0%	0%	6%	0%	0%	0%	14%
Fats and oils	6%	7%	7%	6%	19%	27%	25%	18%
Dairy products (all types)	0%	5%	6%	10%	0%	18%	23%	20%
Nuts and seeds	0%	0%	0%	0%	0%	0%	0%	0%
Potatoes (all types)	0%	0%	0%	0%	1%	2%	1%	1%
Composite meals	0%	0%	0%	0%	1%	1%	0%	0%
Sugar-sweetened beverages	1%	0%	0%	2%	0%	1%	0%	2%
Cereals and grain products	0%	0%	0%	1%	2%	4%	2%	2%
Jams, marmalades, and compotes	0%	0%	0%	0%	0%	0%	0%	0%
Sauces, dressings, and mayonnaise	0%	0%	0%	0%	0%	0%	1%	1%
Sweets and snack foods	4%	5%	1%	1%	19%	8%	7%	9%
Plant-based dairy alternatives	14%	10%	7%	6%	49%	23%	10%	8%
Plant-based meat analogs	0%	0%	0%	0%	3%	0%	0%	0%
Egg	0%	4%	5%	11%	0%	14%	13%	12%
Herbs, spices, condiments	0%	0%	0%	0%	0%	0%	0%	0%
Dietary supplements	72%	68%	64%	47%	0%	0%	0%	0%
Average intake of vitamin D (µg/d)	21.7	18.7	23.5	17.5	4.1	4.4	6.5	7.3

Veg = vegan, LOV = lacto-ovo-vegetarian, Pesc = pescatarian, Omni = omnivore

*Supplement user was defined as having reported any type of vitamin/mineral supplement containing vitamin D in at least one of their 24-hour dietary recalls. Data is shown for descriptive purposes; no statistical tests of differences were conducted. Intakes were not normally distributed. Average intakes from 24-hour dietary recalls (between 2-4 per participant).

Supplementary Table 3 Contribution (%) from food categories and dietary supplements to average intakes of vitamin B_2 (mg/d) among youth, stratified by supplement use and dietary practice (vegan, lacto-ovo-vegetarian, pescatarian, omnivore)

	**Supplement user***				**Non-supplement user**			
	Veg	LOV	Pesc	Omni	Veg	LOV	Pesc	Omni
Number of participants (n)	n=34	n=21	n=13	n=7	n=26	n=38	n=42	n=54
Alcoholic beverages	1%	1%	0%	1%	2%	2%	1%	1%
Legumes	2%	2%	1%	0%	5%	2%	2%	1%
Fish and seafood	0%	0%	1%	1%	0%	0%	2%	2%
Fruit and vegetable juice	0%	0%	0%	0%	0%	1%	0%	0%
Vegetables, fruits, and berries	7%	5%	6%	3%	13%	11%	9%	7%
Coffee and tea	1%	1%	1%	0%	2%	2%	1%	1%
Protein bars/powders	0%	0%	0%	0%	1%	1%	0%	0%
Meat (all types)	0%	0%	0%	9%	0%	0%	0%	18%
Fats and oils	0%	0%	0%	0%	0%	0%	0%	0%
Dairy products (all types)	0%	14%	14%	18%	0%	23%	33%	26%
Nuts and seeds	1%	1%	0%	0%	2%	2%	2%	1%
Potatoes (all types)	1%	1%	1%	1%	2%	1%	2%	2%
Composite meals	1%	1%	0%	0%	1%	1%	1%	0%
Sugar-sweetened beverages	0%	0%	1%	1%	1%	0%	1%	1%
Cereals and grain products	10%	6%	9%	4%	15%	15%	14%	14%
Jams, marmalades, and compotes	0%	0%	0%	0%	0%	0%	0%	0%
Sauces, dressings, and mayonnaise	1%	1%	0%	0%	1%	1%	1%	1%
Sweets and snack foods	3%	5%	4%	6%	12%	8%	13%	11%
Plant-based dairy alternatives	15%	6%	7%	2%	26%	16%	7%	6%
Plant-based meat analogs	7%	6%	6%	0%	11%	6%	5%	1%
Egg	0%	4%	3%	5%	0%	5%	7%	6%
Herbs, spices, condiments	2%	1%	2%	2%	8%	2%	1%	1%
Dietary supplements	47%	47%	43%	46%	0%	0%	0%	0%
Average intake of vitamin B_2 (mg/d)	2.9	3.2	2.9	8.0	1.2	1.3	1.4	1.7

Veg = vegan, LOV = lacto-ovo-vegetarian, Pesc = pescatarian, Omni = omnivore

*Supplement user was defined as having reported any type of vitamin/mineral supplement containing vitamin B_2 in at least one of their 24-hour dietary recalls. Data is shown for descriptive purposes; no statistical tests of differences were conducted. Intakes were not normally distributed. Average intakes from 24-hour dietary recalls (between 2-4 per participant).

Supplementary Table 4 Contribution (%) from food categories and dietary supplements to average intakes of vitamin B_{12} (µg/d) among youth, stratified by supplement use and dietary practice (vegan, lacto-ovo-vegetarian, pescatarian, omnivore)

	Supplement user*				Non-supplement user			
	Veg	LOV	Pesc	Omni	Veg	LOV	Pesc	Omni
Number of participants (n)	n=44	n=25	n=14	n=7	n=16	n=34	n=41	n=54
Alcoholic beverage	0%	0%	0%	0%	0%	0%	0%	0%
Legumes	0%	0%	0%	0%	0%	0%	0%	0%
Fish and seafood	0%	0%	16%	13%	0%	0%	20%	14%
Fruit and vegetable juice	0%	0%	1%	0%	5%	1%	0%	0%
Vegetables, fruits, and berries	0%	0%	0%	0%	0%	2%	0%	0%
Coffee and tea	0%	0%	0%	0%	0%	1%	0%	1%
Protein bars/powders	0%	0%	0%	0%	1%	1%	0%	0%
Meat (all types)	0%	0%	0%	13%	0%	0%	0%	25%
Fats and oils	0%	0%	0%	0%	0%	0%	0%	0%
Dairy products (all types)	0%	9%	13%	23%	0%	43%	46%	33%
Nuts and seeds	0%	0%	0%	0%	0%	0%	0%	0%
Potatoes (all types)	0%	0%	0%	1%	0%	1%	1%	1%
Composite meals	1%	0%	0%	0%	0%	1%	0%	0%
Sugar-sweetened beverages	1%	0%	0%	2%	0%	1%	0%	1%
Cereals and grain products	0%	1%	1%	0%	7%	5%	2%	2%
Jams, marmalades, and compotes	0%	0%	0%	0%	0%	0%	0%	0%
Sauces, dressings, and mayonnaise	0%	0%	0%	0%	0%	1%	1%	1%
Sweets and snack foods	2%	3%	2%	5%	29%	12%	11%	9%
Plant-based dairy alternatives	9%	3%	5%	2%	53%	21%	5%	4%
Plant-based meat analogs	0%	0%	0%	0%	4%	0%	0%	0%
Egg	0%	2%	4%	6%	0%	9%	11%	9%
Herbs, spices, condiments	0%	0%	0%	3%	0%	0%	0%	0%
Dietary supplements	87%	80%	57%	33%	0%	0%	0%	0%
Average intake of vitamin B_{12} (µg/d)	136.1	152.1	26.3	42.8	2.6	2.4	3.9	4.8

Veg = vegan, LOV = lacto-ovo-vegetarian, Pesc = pescatarian, Omni = omnivore

*Supplement user was defined as having reported any type of vitamin/mineral supplement containing vitamin B_{12} in at least one of their 24-hour dietary recalls. Data is shown for descriptive purposes; no statistical tests of differences were conducted. Intakes were not normally distributed. Average intakes from 24-hour dietary recalls (between 2-4 per participant).

Supplementary Table 5 Contribution (%) from food categories and dietary supplements to average intakes of iron (mg/d) among youth, stratified by supplement use and dietary practice (vegan, lacto-ovo-vegetarian, pescatarian, omnivore)

	**Supplement user***				**Non-supplement user**			
	Veg	LOV	Pesc	Omni	Veg	LOV	Pesc	Omni
Number of participants (n)	n=35	n=23	n=13	n=5	n=25	n=36	n=42	n=56
Alcoholic beverages	0%	1%	0%	1%	2%	1%	2%	1%
Legumes	8%	7%	6%	2%	15%	8%	7%	2%
Fish and seafood	0%	0%	1%	0%	0%	0%	2%	1%
Fruit and vegetable juice	0%	0%	0%	0%	0%	1%	0%	0%
Vegetables, fruits, and berries	6%	6%	9%	7%	12%	14%	12%	12%
Coffee and tea	0%	0%	0%	0%	1%	0%	0%	0%
Protein bars/powders	1%	1%	0%	0%	1%	0%	1%	1%
Meat (all types)	0%	0%	0%	14%	0%	0%	0%	19%
Fats and oils	0%	0%	0%	0%	0%	0%	0%	0%
Dairy products (all types)	0%	1%	1%	1%	0%	2%	2%	1%
Nuts and seeds	3%	2%	2%	2%	5%	5%	5%	4%
Potatoes (all types)	1%	1%	2%	2%	2%	4%	4%	3%
Composite meals	2%	0%	0%	1%	2%	1%	1%	0%
Sugar-sweetened beverages	0%	0%	0%	0%	1%	1%	1%	0%
Cereals and grain products	14%	11%	14%	18%	22%	28%	30%	27%
Jams, marmalades, and compotes	0%	0%	0%	0%	0%	0%	0%	0%
Sauces, dressings, and mayonnaise	1%	1%	1%	0%	1%	1%	1%	2%
Sweets and snack foods	7%	7%	6%	4%	9%	8%	11%	14%
Plant-based dairy alternatives	2%	1%	1%	0%	4%	2%	1%	1%
Plant-based meat analogs	12%	12%	7%	0%	19%	16%	8%	3%
Egg	0%	2%	2%	3%	0%	3%	5%	4%
Herbs, spices, condiments	3%	2%	3%	1%	6%	5%	4%	4%
Dietary supplements	39%	44%	43%	43%	0%	0%	0%	0%
Average intake of iron (mg/d)	24.2	23.9	25.7	22.5	13.4	10.6	10.0	11.9

Veg = vegan, LOV = lacto-ovo-vegetarian, Pesc = pescatarian, Omni = omnivore

*Supplement user was defined as having reported any type of vitamin/mineral supplement containing iron in at least one of their 24-hour dietary recalls. Data is shown for descriptive purposes; no statistical tests of differences were conducted. Intakes were not normally distributed. Average intakes from 24-hour dietary recalls (between 2-4 per participant).

Supplementary Table 6 Contribution (%) from food categories and dietary supplements to average intakes of calcium (mg/d) among youth, stratified by supplement use and dietary practice (vegan, lacto-ovo-vegetarian, pescatarian, omnivore) (n=235)

	**Supplement user***				**Non-supplement user**			
	Veg	LOV	Pesc	Omni	Veg	LOV	Pesc	Omni
Number of participants (n)	n=31	n=19	n=12	n=3	n=29	n=40	n=43	n=58
Alcoholic beverage	0%	1%	0%	0%	1%	1%	1%	1%
Legumes	6%	3%	2%	0%	8%	3%	2%	1%
Fish and seafood	0%	0%	1%	0%	0%	0%	1%	1%
Fruit and vegetable juice	1%	0%	0%	0%	0%	0%	0%	0%
Vegetables, fruits, and berries	11%	8%	10%	11%	16%	10%	8%	9%
Coffee and tea	1%	1%	1%	0%	2%	1%	1%	1%
Protein bars/powders	1%	1%	1%	2%	1%	0%	0%	0%
Meat (all types)	0%	0%	0%	2%	0%	0%	0%	3%
Fats and oils	0%	0%	0%	0%	0%	0%	0%	0%
Dairy products (all types)	0%	36%	40%	37%	0%	38%	53%	48%
Nuts and seeds	2%	2%	2%	4%	3%	2%	1%	2%
Potatoes (all types)	1%	1%	2%	0%	1%	1%	1%	2%
Composite meals	1%	0%	0%	2%	2%	1%	0%	0%
Sugar-sweetened beverages	1%	0%	0%	0%	1%	1%	1%	1%
Cereals and grain products	8%	6%	6%	5%	11%	9%	9%	9%
Jams, marmalades, and compotes	0%	0%	0%	0%	0%	0%	0%	0%
Sauces, dressings, and mayonnaise	1%	1%	1%	0%	1%	1%	1%	1%
Sweets and snack foods	5%	6%	6%	6%	8%	6%	8%	9%
Plant-based dairy alternatives	25%	8%	10%	3%	26%	15%	5%	6%
Plant-based meat analogs	14%	8%	7%	1%	16%	8%	4%	1%
Egg	0%	2%	2%	2%	0%	1%	1%	2%
Herbs, spices, condiments	3%	1%	1%	2%	3%	2%	2%	2%
Dietary supplements	19%	14%	7%	19%	0%	0%	0%	0%
Average intake of calcium (mg/d)	800	1142	993	1011	620	914	978	918

Veg = vegan, LOV = lacto-ovo-vegetarian, Pesc = pescatarian, Omni = omnivore

*Supplement user was defined as having reported any type of vitamin/mineral supplement containing calcium in at least one of their 24-hour dietary recalls. Data is shown for descriptive purposes; no statistical tests of differences were conducted. Intakes were not normally distributed. Average intakes from 24-hour dietary recalls (between 2-4 per participant).

Supplementary Table 7 Contribution (%) from food categories and dietary supplements to average intakes of selenium (µg/d) among youth, stratified by supplement use and dietary practice (vegan, lacto-ovo-vegetarian, pescatarian, omnivore)

	**Supplement user***				**Non-supplement user**			
	Veg	LOV	Pesc	Omni	Veg	LOV	Pesc	Omni
Number of participants (n)	n=30	n=20	n=11	n=5	n=30	n=39	n=44	n=56
Alcoholic beverages	0%	0%	0%	0%	0%	0%	0%	0%
Legumes	12%	5%	6%	1%	23%	10%	6%	1%
Fish and seafood	0%	0%	14%	3%	0%	0%	18%	14%
Fruit and vegetable juice	0%	0%	0%	0%	0%	0%	0%	0%
Vegetables, fruits, and berries	4%	3%	4%	2%	7%	8%	6%	4%
Coffee and tea	0%	0%	0%	0%	1%	1%	0%	0%
Protein bars/powders	1%	1%	0%	0%	1%	1%	0%	0%
Meat (all types)	0%	0%	0%	18%	0%	0%	0%	30%
Fats and oils	0%	0%	0%	0%	0%	0%	0%	0%
Dairy products (all types)	0%	8%	10%	13%	0%	18%	19%	14%
Nuts and seeds	4%	3%	2%	0%	7%	7%	5%	3%
Potatoes (all types)	1%	0%	0%	0%	1%	2%	1%	1%
Composite meals	1%	0%	0%	1%	2%	1%	0%	0%
Sugar-sweetened beverages	1%	0%	0%	2%	0%	0%	0%	0%
Cereals and grain products	10%	9%	7%	6%	23%	20%	16%	14%
Jams, marmalades, and compotes	0%	0%	0%	0%	0%	0%	0%	0%
Sauces, dressings, and mayonnaise	0%	1%	0%	0%	1%	1%	1%	1%
Sweets and snack foods	2%	2%	3%	5%	9%	5%	5%	5%
Plant-based dairy alternatives	0%	0%	0%	0%	1%	1%	0%	0%
Plant-based meat analogs	10%	8%	6%	1%	22%	12%	6%	1%
Egg	0%	9%	8%	9%	0%	11%	15%	12%
Herbs, spices, condiments	0%	0%	0%	0%	2%	1%	1%	0%
Dietary supplements	52%	50%	39%	38%	0%	0%	0%	0%
Average intake of selenium (µg/d)	63	71	61	85	26	29	41	49

Veg = vegan, LOV = lacto-ovo-vegetarian, Pesc = pescatarian, Omni = omnivore

*Supplement user was defined as having reported any type of vitamin/mineral supplement containing selenium in at least one of their 24-hour dietary recalls. Data is shown for descriptive purposes; no statistical tests of differences were conducted. Intakes were not normally distributed. Average intakes from 24-hour dietary recalls (between 2-4 per participant).

Supplementary Table 8 Contribution (%) from food categories and dietary supplements to average intakes of zinc (mg/d) among youth, stratified by supplement use and dietary practice (vegan, lacto-ovo-vegetarian, pescatarian, omnivore)

	Supplement user*				Non-supplement user			
	Veg	LOV	Pesc	Omni	Veg	LOV	Pesc	Omni
Number of participants (n)	n=33	n=22	n=11	n=7	n=27	n=37	n=44	n=54
Alcoholic beverages	0%	0%	0%	0%	0%	0%	0%	0%
Legumes	6%	5%	4%	1%	12%	5%	4%	1%
Fish and seafood	0%	0%	2%	1%	0%	0%	3%	2%
Fruit and vegetable juice	0%	0%	0%	0%	0%	0%	0%	0%
Vegetables, fruits, and berries	5%	4%	4%	4%	10%	9%	7%	6%
Coffee and tea	0%	0%	1%	0%	1%	1%	0%	1%
Protein bars/powders	1%	1%	0%	0%	1%	1%	1%	0%
Meat (all types)	0%	0%	0%	15%	0%	0%	0%	31%
Fats and oils	0%	0%	0%	0%	0%	0%	0%	0%
Dairy products (all types)	0%	11%	13%	11%	0%	20%	27%	17%
Nuts and seeds	5%	3%	2%	2%	8%	6%	6%	4%
Potatoes (all types)	1%	1%	1%	1%	2%	2%	2%	2%
Composite meals	1%	1%	0%	1%	2%	1%	1%	0%
Sugar-sweetened beverages	0%	0%	0%	1%	0%	0%	0%	1%
Cereals and grain products	17%	13%	15%	12%	30%	28%	27%	23%
Jams, marmalades, and compotes	0%	0%	0%	0%	0%	0%	0%	0%
Sauces, dressings, and mayonnaise	0%	1%	1%	0%	1%	1%	1%	1%
Sweets and snack foods	4%	4%	4%	5%	8%	6%	7%	7%
Plant-based dairy alternatives	2%	1%	1%	0%	4%	2%	1%	1%
Plant-based meat analogs	12%	10%	13%	1%	18%	13%	8%	1%
Egg	0%	2%	2%	2%	0%	2%	4%	3%
Herbs, spices, condiments	1%	0%	0%	1%	2%	1%	1%	1%
Dietary supplements	43%	43%	36%	42%	0%	0%	0%	0%
Average intake of zinc (mg/d)	15.7	17.5	15.7	23.1	8.0	8.7	9.1	11.7

Veg = vegan, LOV = lacto-ovo-vegetarian, Pesc = pescatarian, Omni = omnivore

*Supplement user was defined as having reported any type of vitamin/mineral supplement containing zinc in at least one of their 24-hour dietary recalls. Data is shown for descriptive purposes; no statistical tests of differences were conducted. Intakes were not normally distributed. Average intakes from 24-hour dietary recalls (between 2-4 per participant).

Supplementary Table 9 Contribution (%) from animal-sourced foods (dairy products, eggs, fish and seafood, meat [all types]) to average intakes of energy, protein, saturated fatty acids, and micronutrients among vegans, lacto-ovo-vegetarians, pescatarians, and omnivores aged 16-24 years old (n=235)

	Vegan (n=60)	Lacto-ovo-vegetarian (n=59)	Pescatarian (n=55)	Omnivore (n=61)
Energy	0%	14%	20%	28%
Protein	0%	25%	39%	58%
Saturated fatty acids	0%	39%	46%	53%
Vitamin A	0%	30%	38%	42%
Vitamin D	0%	31%	53%	58%
Vitamin E	0%	10%	19%	26%
Vitamin K	0%	12%	18%	27%
Vitamin B_1	0%	6%	13%	32%
Vitamin B_2	0%	30%	40%	52%
Vitamin C	0%	1%	2%	5%
Niacin	0%	18%	31%	54%
Vitamin B_6	0%	6%	14%	34%
Vitamin B_{12}	0%	57%	75%	80%
Folate	0%	11%	17%	25%
Phosphorous	0%	25%	36%	48%
Iron	0%	5%	9%	26%
Calcium	0%	41%	53%	53%
Potassium	0%	9%	16%	29%
Magnesium	0%	8%	14%	23%
Selenium	0%	32%	52%	69%
Zinc	0%	23%	32%	51%

Data is shown for descriptive purposes; no statistical tests of differences were conducted.

Supplementary Table 10 Contribution (%) from plant-based meat analogs and plant-based dairy alternatives to average intakes of energy, protein, saturated fatty acids, and micronutrients among vegans, lacto-ovo-vegetarians, pescatarians, and omnivores aged 16-24 years old (n=235)

	Vegan (n=60)	Lacto-ovo-vegetarian (n=59)	Pescatarian (n=55)	Omnivore (n=61)
Energy	18%	10%	6%	2%
Protein	30%	18%	11%	2%
Saturated fatty acids	32%	11%	6%	3%
Vitamin A	4%	2%	1%	1%
Vitamin D	51%	27%	13%	8%
Vitamin E	23%	16%	9%	4%
Vitamin K	8%	5%	2%	1%
Vitamin B_1	22%	18%	12%	3%
Vitamin B_2	40%	23%	14%	7%
Vitamin C	2%	2%	1%	0%
Niacin	16%	10%	6%	1%
Vitamin B_6	17%	13%	7%	3%
Vitamin B_{12}	66%	19%	7%	4%
Folate	25%	19%	12%	4%
Phosphorous	29%	18%	10%	4%
Iron	23%	20%	11%	3%
Calcium	45%	22%	11%	7%
Potassium	17%	12%	7%	3%
Magnesium	24%	18%	11%	3%
Zinc	23%	17%	11%	2%
Selenium	23%	14%	7%	1%

Data is shown for descriptive purposes; no statistical tests of differences were conducted.

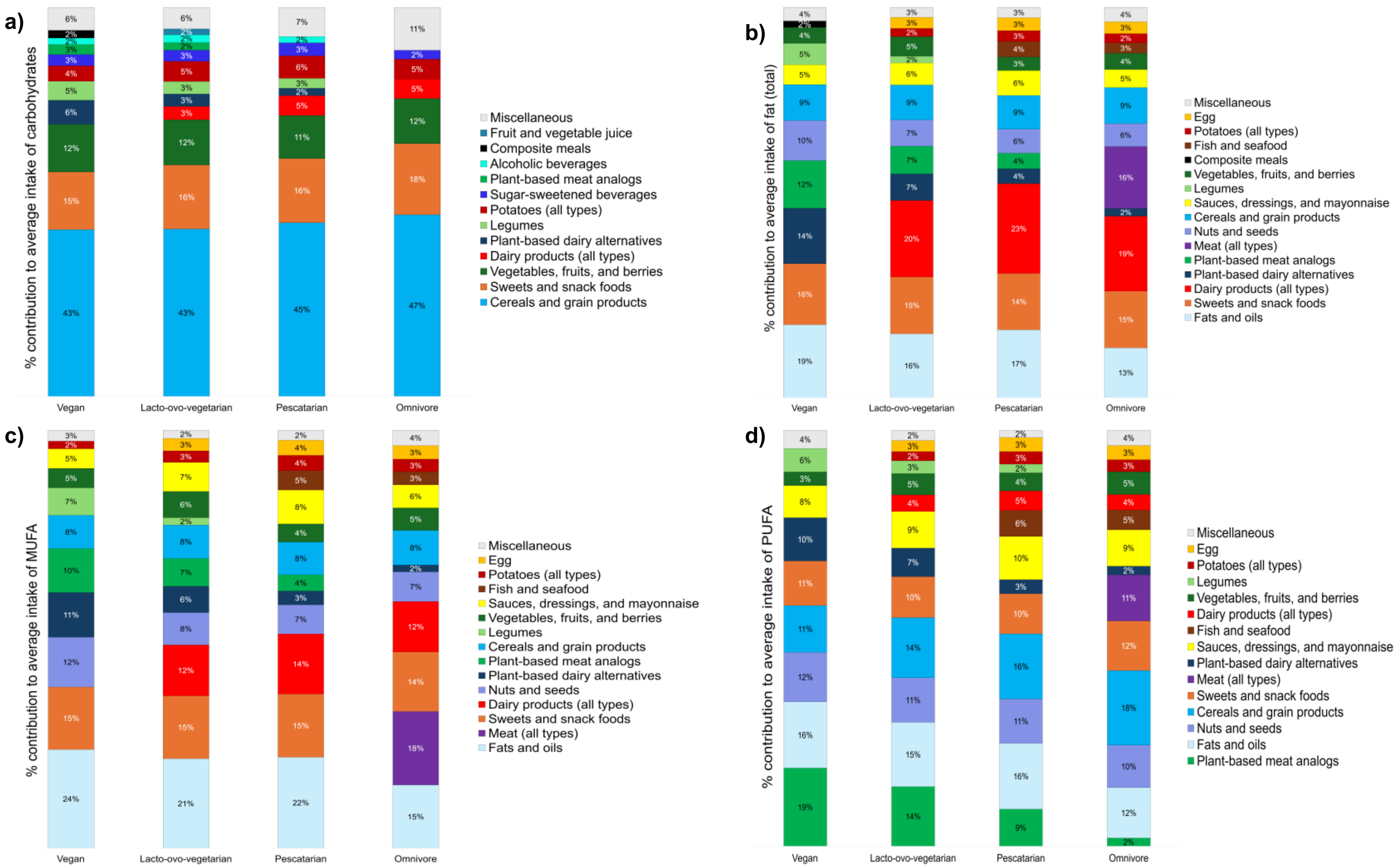

Supplementary Fig 1 The contribution (%) of different food categories to the average intakes of a) carbohydrates, b) total fat, c) monounsaturated fatty acids (MUFA), and d) polyunsaturated fatty acids (PUFA) among youth adhering to vegan, lacto-ovo-vegetarian, pescatarian, or omnivorous diets. The category ´miscellaneous´ includes all food categories that each contributed ≤1% of the average intake, and represents the sum of these categories

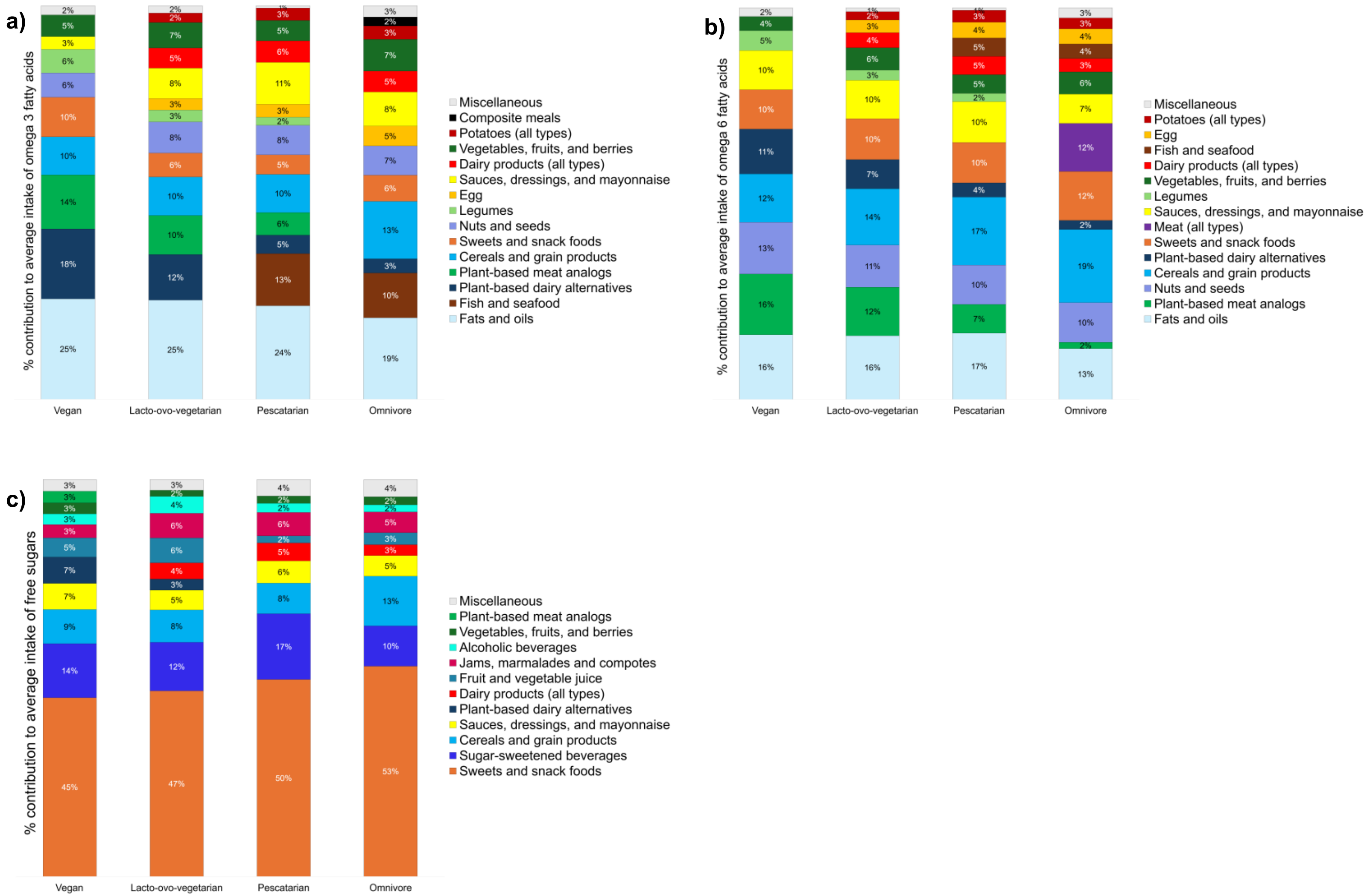

Supplementary Fig 2 The contribution (%) of different food categories to the average intakes of a) omega-3 fatty acids, b) omega-6 fatty acids, and c) free sugars among youth adhering to vegan, lacto-ovo-vegetarian, pescatarian, or omnivorous diets. The category ´miscellaneous´ includes all food categories that each contributed ≤1% of the average intake, and represents the sum of these categories. Omega-3 fatty acids include the fatty acids EPA, DHA, and DPA. Omega-6 fatty acids include the fatty acids LA and AA

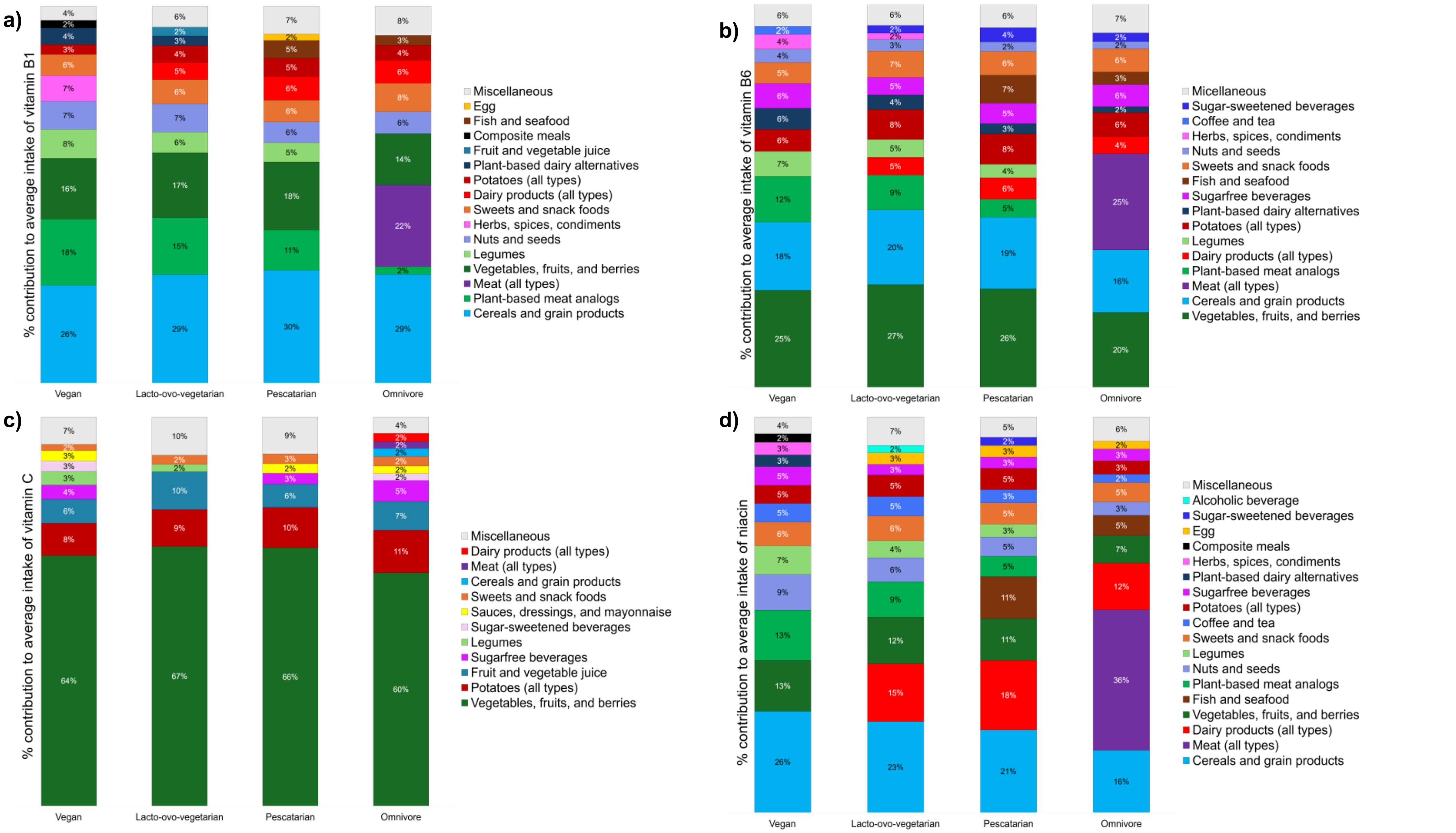

Supplementary Fig 3 The contribution (%) of different food categories to the average intakes of a) vitamin B_1, b) vitamin B_6, c) vitamin C, and d) niacin among youth adhering to vegan, lacto-ovo-vegetarian, pescatarian, or omnivorous diets. The category ´miscellaneous´ includes all food categories that each contributed ≤1% of the average intake, and represents the sum of these categories. The category ´herbs, spices, condiments´ includes nutritional yeast, a food rich in some B-vitamins

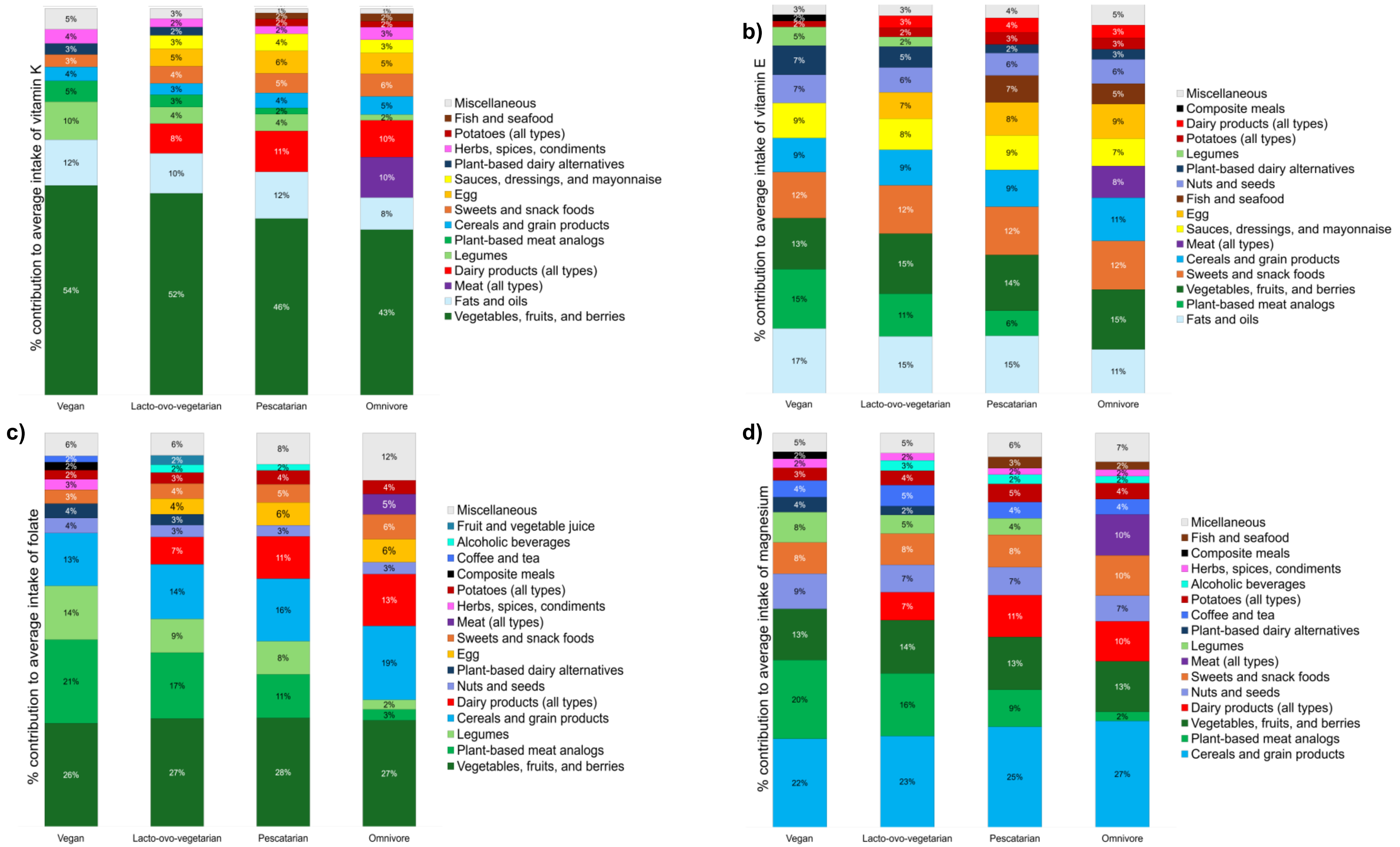

Supplementary Fig 4 The contribution (%) of different food categories to the average intakes of a) vitamin K, b) vitamin E, c) folate, and d) magnesium among youth adhering to vegan, lacto-ovo-vegetarian, pescatarian, or omnivorous diets. The category ´miscellaneous´ includes all food categories that each contributed ≤1% of the average intake, and represents the sum of these categories

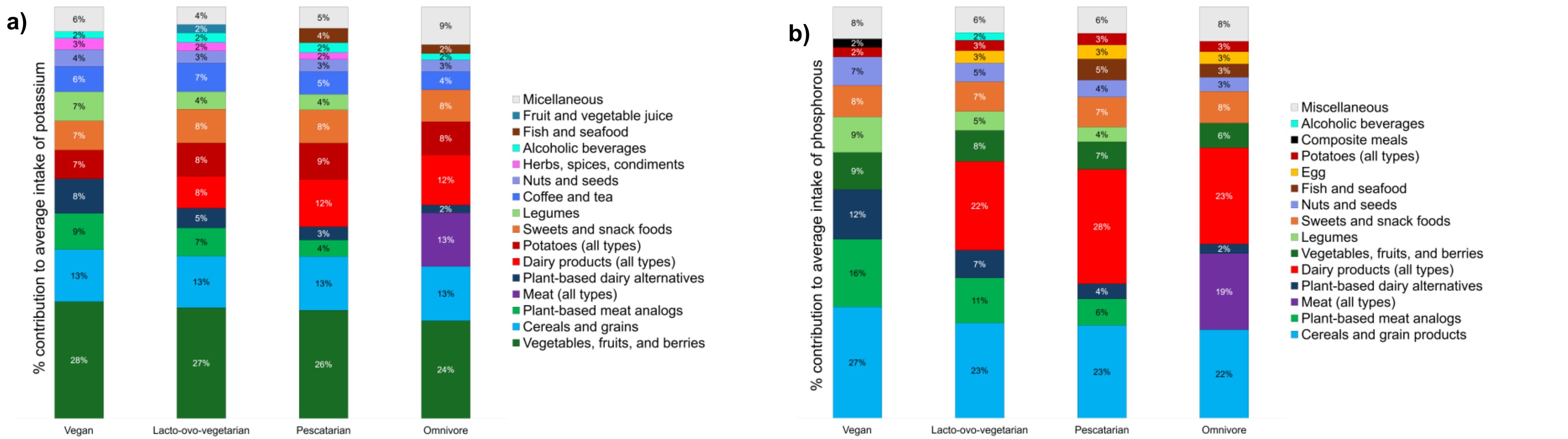

Supplementary Fig 5 The contribution (%) of different food categories to the average intakes of a) potassium and b) phosphorous among youth adhering to vegan, lacto-ovo-vegetarian, pescatarian, or omnivorous diets. The category ´miscellaneous´ includes all food categories that each contributed ≤1% of the average intake, and represents the sum of these categories

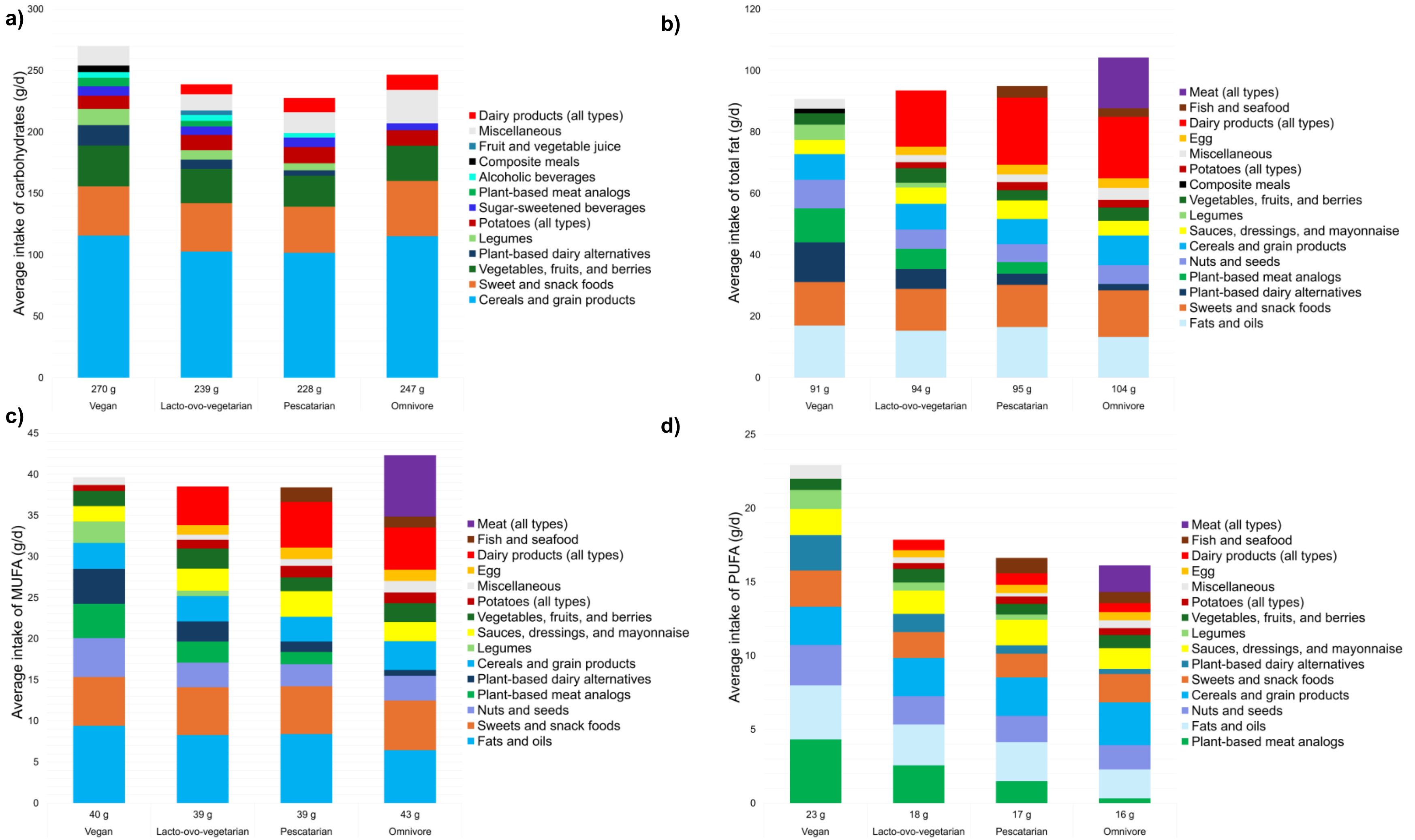

Supplementary Fig 6 The contribution of different food categories relative to the average intake of a) carbohydrates, b) total fat, c) monounsaturated fatty acids (MUFA), and d) polyunsaturated fatty acids (PUFA) among youth adhering to vegan, lacto-ovo-vegetarian, pescatarian, or omnivorous diets. The category ´miscellaneous´ includes all food categories that each contributed ≤1% of the average intake, and represents the sum of these categories. Values shown below each stacked bar indicate the total average intake of the nutrient from food sources only

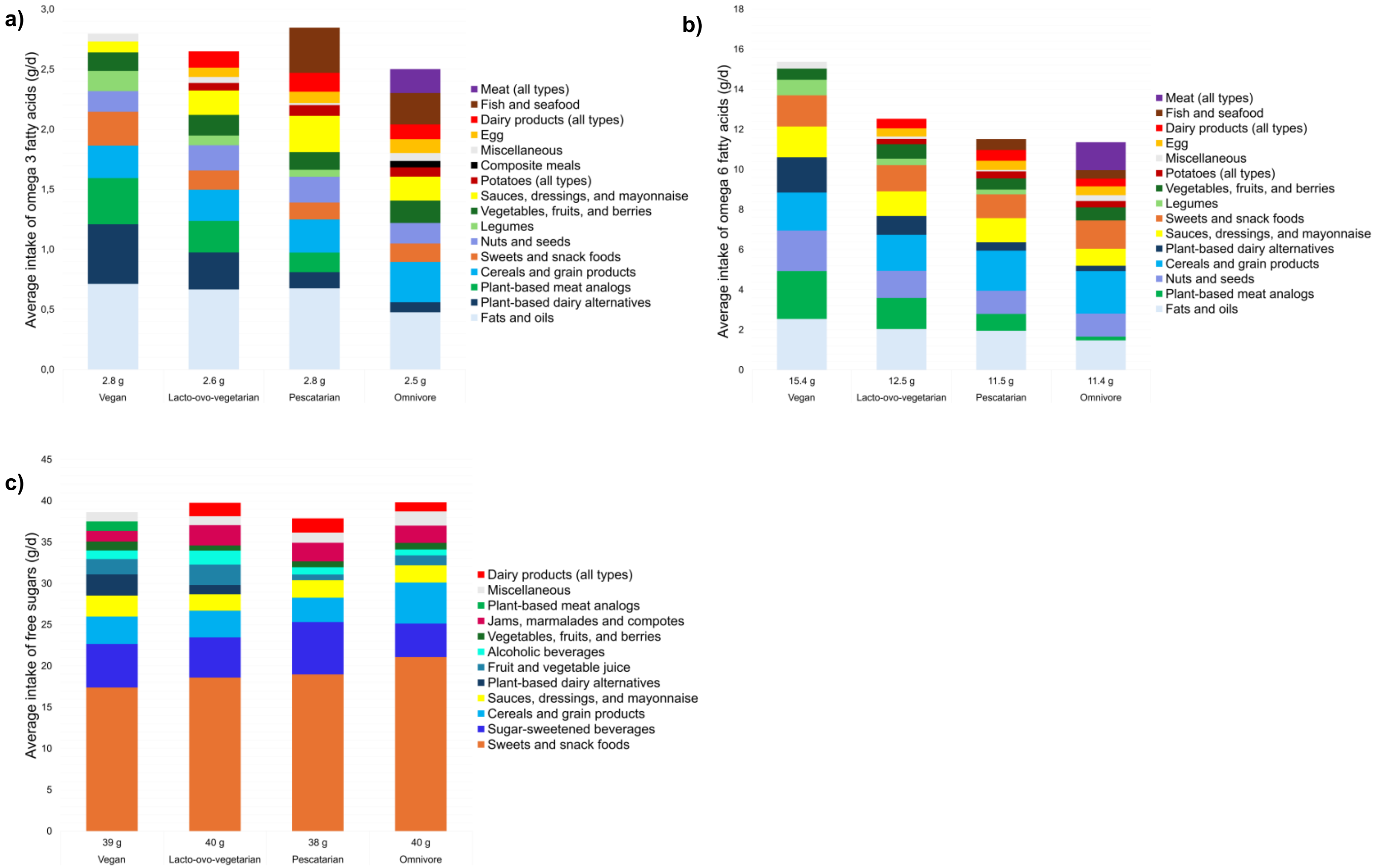

Supplementary Fig 7 The contribution of different food categories relative to the average intake of a) omega-3 fatty acids, b) omega-6 fatty acids, and c) free sugars among youth adhering to vegan, lacto-ovo-vegetarian, pescatarian, or omnivorous diets. The category ´miscellaneous´ includes all food categories that each contributed ≤1% of the average intake, and represents the sum of these categories. Omega-3 fatty acids include the fatty acids EPA, DHA and DPA. Omega-6 fatty acids include the fatty acids LA and AA. Values shown below each stacked bar indicate the total average intake of the nutrient from food sources only

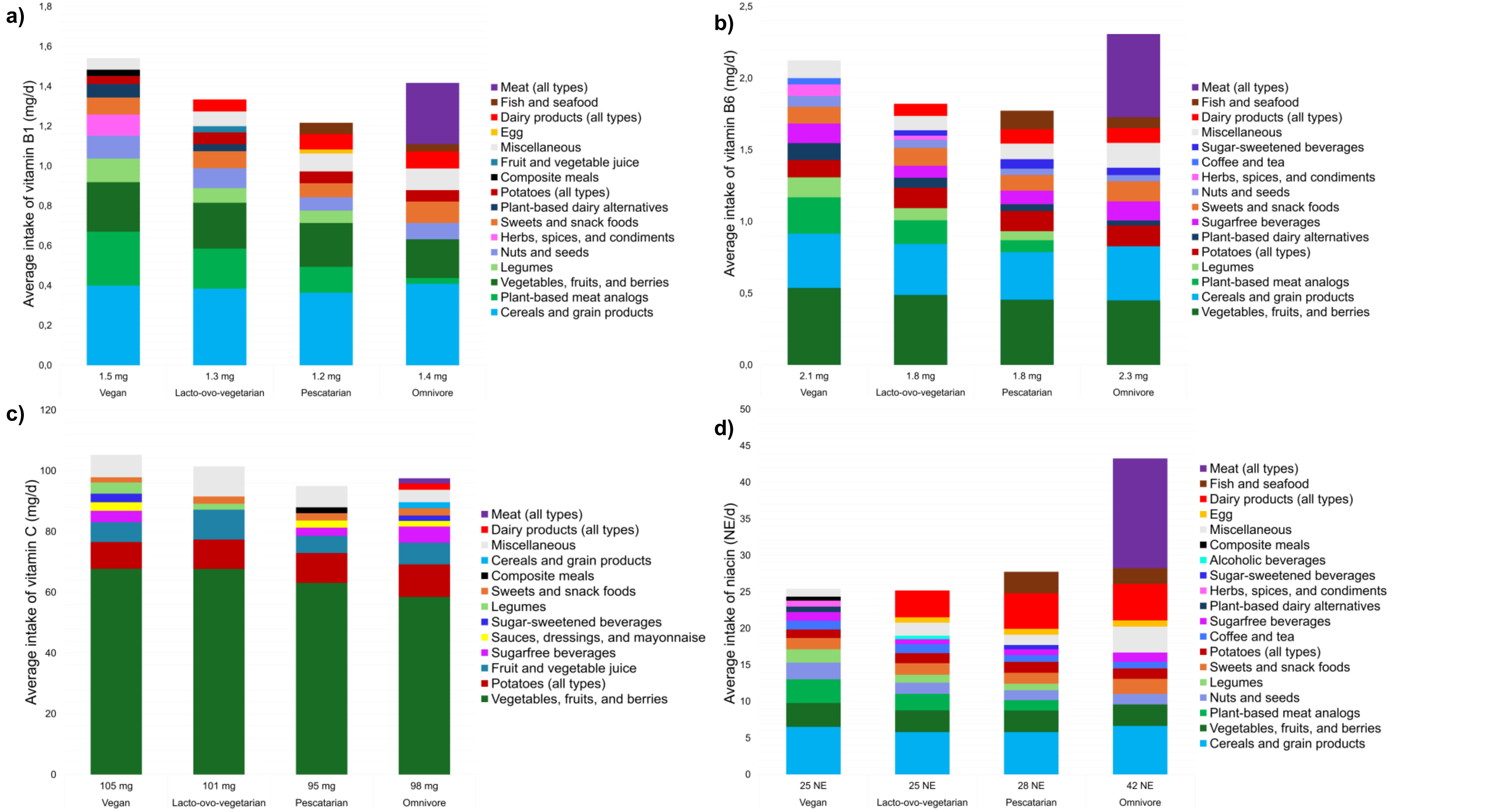

Supplementary Fig 8 The contribution of different food categories relative to the average intake of a) vitamin B_1, b) vitamin B_6, c) vitamin C, and d) niacin among youth adhering to vegan, lacto-ovo-vegetarian, pescatarian, or omnivorous diets. The category ´miscellaneous´ includes all food categories that each contributed ≤1% of the average intake, and represents the sum of these categories. Values shown below each stacked bar indicate the total average intake of the nutrient from food sources only

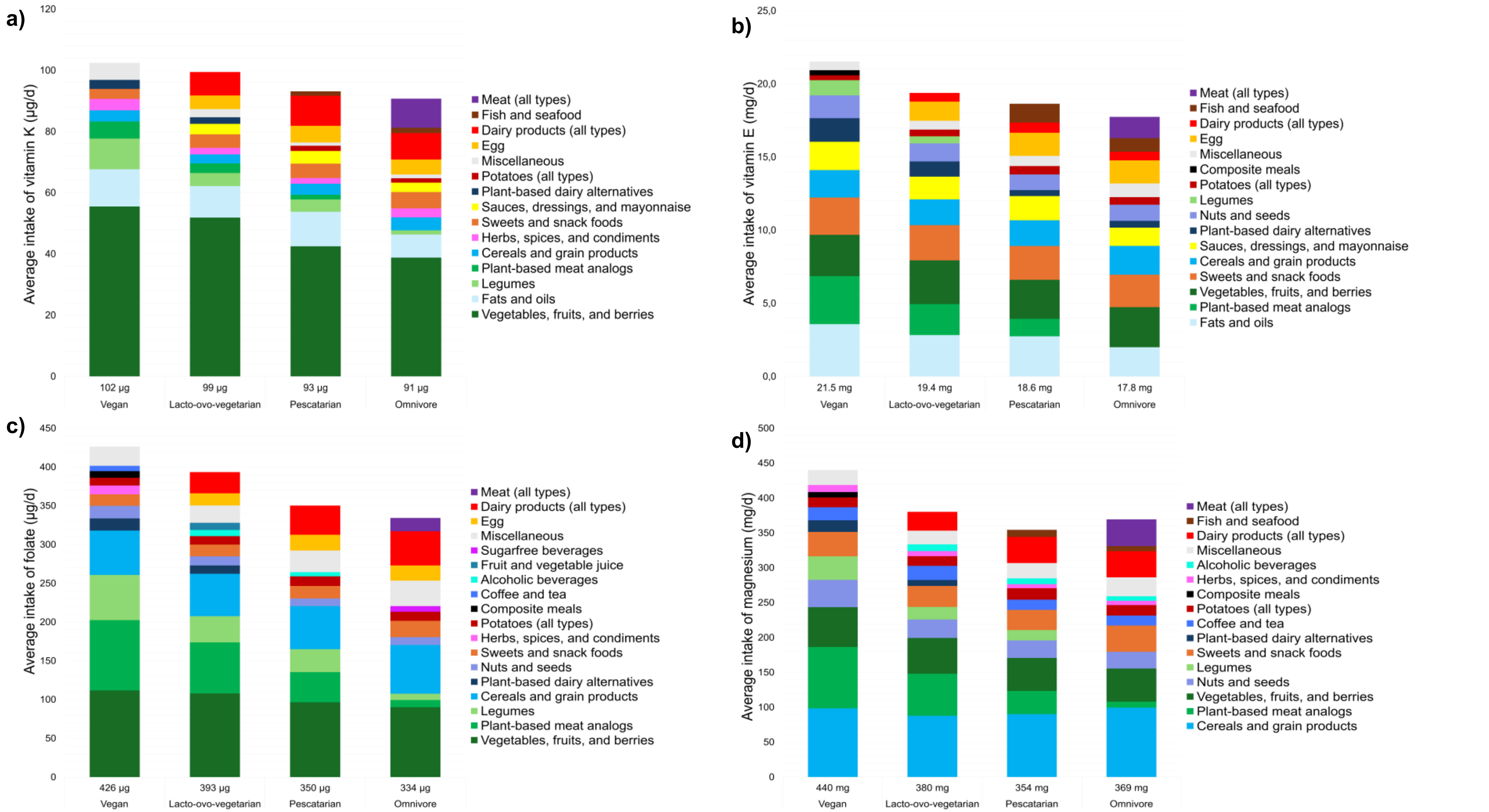

Supplementary Fig 9 The contribution of different food categories relative to the average intake of a) vitamin K, b) vitamin E, c) folate, and d) magnesium among youth adhering to vegan, lacto-ovo-vegetarian, pescatarian, or omnivorous diets. The category ´miscellaneous´ includes all food categories that each contributed ≤1% of the average intake, and represents the sum of these categories. Values shown below each stacked bar indicate the total average intake of the nutrient from food sources only

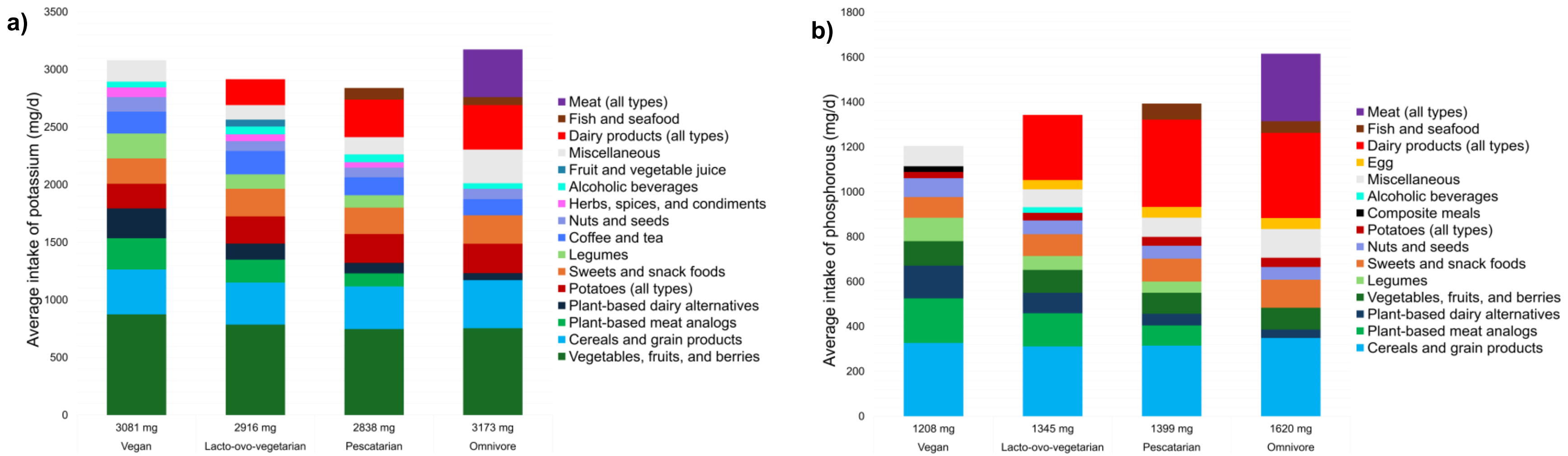

Supplementary Fig 10 The contribution of different food categories relative to the average intake of a) potassium and b) phosphorous among youth adhering to vegan, lacto-ovo-vegetarian, pescatarian, or omnivorous diets. The category ´miscellaneous´ includes all food categories that each contributed ≤1% of the average intake, and represents the sum of these categories. Values shown below each stacked bar indicate the total average intake of the nutrient from food sources only
